# Cardiomyocyte-derived small extracellular vesicle: a new mechanism driving diabetic cardiac fibrosis and cardiomyopathy

**DOI:** 10.7150/thno.99507

**Published:** 2024-09-09

**Authors:** Yu Li, Yunhui Du, Yang Liu, Xiuhuan Chen, Xinxin Li, Yanru Duan, Yanwen Qin, Huirong Liu, Xinliang Ma, Shaoping Nie, Huina Zhang

**Affiliations:** 1 Department of Cardiology, Beijing An Zhen Hospital, Capital Medical University, Beijing, 100029, China.; 2 Beijing An Zhen Hospital, Capital Medical University, The Key Laboratory of Remodeling Cardiovascular Diseases, Ministry of Education; Collaborative Innovation Center for Cardiovascular Disorders, Beijing Institute of Heart Lung and Blood Vessel Disease, Beijing, 100029, China.; 3 Department of Physiology & Pathophysiology, School of Basic Medical Sciences, Capital Medical University, Beijing, 100029, China.; 4 Department of Emergency Medicine, Thomas Jefferson University, Philadelphia, PA, PA19107, USA.

**Keywords:** Diabetic cardiomyopathy, Cardiac fibrosis, Small extracellular vesicles, miRNA

## Abstract

**Rationale:** Diabetic cardiomyopathy is one of the major diabetic cardiovascular complications in which fibrosis plays a critical pathogenetic role. However, the precise mechanisms by which diabetes triggers cardiac fibrosis in the heart remain elusive. Small extracellular vesicles (sEVs) play an important role in the cellular communication. Nevertheless, whether and how diabetes may adversely alter sEVs-mediated cardiomyocyte-fibroblast communication, promoting diabetic cardiac fibrosis and contributing to diabetic cardiomyopathy, has not been previously investigated.

**Methods and results:** High-fat diet (HFD)-induced and genetic (*db/db*) type 2 diabetic models were utilized. Cardiomyocyte sEVs (Myo-sEVs) were isolated by ultracentrifugation. Normal cardiomyocyte-derived Myo-sEVs attenuated diabetic cardiac fibrosis *in vitro* and *in vivo* and improved cardiac diastolic function. In contrast, diabetic cardiomyocyte-derived Myo-sEVs significantly exacerbated diabetic cardiac fibrosis and worsened diastolic function. Unbiased miRNA screening analysis revealed that miR-194-3p was significantly reduced in diabetic Myo-sEVs. Additional *in vitro* and *in vivo* experiments demonstrated that miR-194-3p is a novel upstream molecule inhibiting TGFβR2 expression and blocking fibroblast-myofibroblast conversion. Administration of miR-194-3p mimic or agomiR-194-3p significantly reduced diabetic cardiac fibrosis *in vitro* and *in vivo*, and attenuated diabetic cardiomyopathy.

**Conclusion:** Our study demonstrates for the first time that cardiomyocyte-derived miR194-3p inhibits TGFβ-mediated fibroblast-to-myofibroblast conversion, acting as an internal break against cardiac fibrosis. Diabetic downregulation of sEV-mediated miR-194-3p delivery from cardiomyocytes to fibroblasts contributes to diabetic cardiac fibrosis and diabetic cardiomyopathy. Pharmacological or genetic restoration of this system may be a novel therapy against diabetic cardiomyopathy.

## Introduction

Diabetes mellitus (DM) and its associated complications represent a global burden on human health and economics. Cardiovascular diseases are the leading cause of death in diabetic patients, with a 2-5 times higher risk of developing heart failure than in age-matched non-diabetic patients 1. Diabetic cardiomyopathy, one of the major diabetic cardiovascular complications, is defined as the presence of abnormal myocardial structure and function in individuals with diabetes mellitus, independent of any other cardiac risk factors 2. In its early stages, diabetic cardiomyopathy involves a latent subclinical phase characterized by structural and functional abnormalities, including left ventricular hypertrophy, fibrosis, and cell signaling abnormalities 2. The pathogenesis and clinical features of diabetic cardiomyopathy have been well studied over the past decade, but effective approaches to prevent and treat this disease are limited. Clarifying the mechanisms and identifying effective targets in the early stage of diabetic cardiomyopathy is an urgent unmet medical need.

Diabetic cardiac fibrosis is a major pathological contributor to diabetic cardiomyopathy, characterized by excessive deposition of collagen fibers in the heart, leading to impaired left ventricular diastolic function, eventually to cardiac dysfunction and heart failure with high morbidity and mortality worldwide 2. Extracellular matrix deposition by activated myofibroblasts in response to fibrogenic cytokines is the hallmark of cardiac remodeling in diabetic cardiomyopathy 3. The transforming growth factor β (TGFβ) signaling pathway plays a significant role in the process of diabetic cardiac fibrosis and diabetic cardiomyopathy 4. During the process of myocardial fibrosis, TGFβ binds to TGFβ receptor 2 (TGFβR2), which recruits TGFβ receptor 1 (TGFβR1) and causes its phosphorylation, thereby activating downstream canonical Smad pathways and non-canonical pathways such as TAK1, p38 MAPK, and JNK, which are collectively involved in the process of myocardial fibrosis^4^. However, there are still no effective clinical therapeutic strategies to directly control the onset and progression of diabetic cardiac fibrosis by directly targeting this pathway 5. Recent evidence suggests that impaired endogenous defense mechanisms are a major cause of diabetic cardiomyopathy 6. Therefore, the identification of internal braking factors that inhibit diabetic cardiac fibrosis is essential for the effective treatment of diabetic cardiomyopathy.

Small extracellular vesicles (sEVs) play an important role in the cellular microenvironment by transferring proteins, nucleic acids, and lipids to facilitate cell communication 7. They have either harmful or beneficial effects on cardiac remodeling, depending on the contents they contain 8. Abnormal augment of endogenous harmful factors or reduction of endogenous protective factors in sEVs contributes to pathological cardiac phenotype. For instance, macrophage-derived sEVs containing increased miR-155 promote inflammation following cardiac injury 9. sEVs with elevated miR-130b-3p delivered from dysfunctional adipocytes significantly exacerbate myocardial ischemia/reperfusion injury in diabetic mice 10. In contrast, sEVs from induced pluripotent stem cells or cardiac progenitor cells aid in cardiac repair by transferring endogenous protective molecules 11, 12. Cardiomyocytes are important cells that maintain a healthy cardiac microenvironment 13. A recent study demonstrated that cardiomyocyte-derived sEVs (Myo-sEVs) from healthy donor hearts exert significant cardioprotection against post-myocardial infarction remodeling, but Myo-sEVs from heart failure patients are detrimental due to reduced pro-angiogenic molecules such as miR-21-5p 14. Nevertheless, whether and how diabetes may adversely alter sEVs-mediated cardiomyocyte-fibroblast communication, promoting diabetic cardiac fibrosis and contributing to diabetic cardiomyopathy, has not been previously investigated.

The current study aims to 1) determine the role of Myo-sEVs in diabetic cardiac fibrosis, 2) identify the critical endogenous protective/anti-fibrosis miRNAs that are reduced in diabetic Myo-sEVs, and 3) discover the mechanism by which diabetic Myo-sEV miRNA mediates the phenotype conversion of fibroblasts to myofibroblasts. This study could expand the scope of cardiomyocyte-borne substances involved in diabetic cardiac fibrosis and help identify new therapeutic targets for this epidemic condition.

## Methods

The data that support the findings of this study are available from the corresponding author upon reasonable request. Detailed methods are available in the Data Supplement.

### Clinical samples

The study design and protocol were approved by the Medical Ethics Committee of Beijing Anzhen Hospital (2022036) and strictly followed the guidelines of the Declaration of Helsinki. 30 patients with type 2 diabetes mellitus (T2DM) and 30 healthy controls (non-diabetes) were recruited from the Department of Endocrinology and Health Management Center of Beijing AnZhen Hospital from June to November 2022 (Table S1). Written informed consent was obtained from all participants. Clinical test data were retrieved from hospital databases and analyzed. The diagnosis of T2DM in patients was based on the American Diabetes Association criteria 15, which included a previous diagnosis of T2DM, fasting blood glucose ≥ 7mM, or 2-hour plasma glucose ≥ 11.1 mM during 75 g oral glucose tolerance test, or random glucose concentrations ≥ 11.1 mM, or HbA1c ≥ 6.5%, or patients receiving glucose-lowering treatment for diabetes.

### Animal studies

All animal experiments were approved by the Capital Medical University Animal Experimentation Ethics Committee, and in compliance with the National Institutes of Health Guidelines on the Use of Laboratory Animals. High-fat diet (HFD)-induced 16 and genetic (*db/db*) type 2 diabetic models were utilized in this study. Primary cardiomyocytes and primary fibroblasts were obtained from the hearts of adult mice after anesthesia with 2% isoflurane followed by cervical dislocation.

### Measurement of blood pressure by tail-cuff plethysmography

The Softron BP2010A system (Beijing, China) for non-invasive monitoring of blood pressure in mice was utilized to assess blood pressure by tracking changes in tail volume.

### Isolation and culture of cardiomyocyte

Primary cardiomyocytes from C57BL/6 male mice were isolated following a previously established protocol 17.

### Isolation and culture of cardiac fibroblast

Primary cardiac fibroblasts from C57BL/6 male mice were isolated as previously described 18.

### Glucose and insulin tolerance tests

To assess glucose tolerance, mice were intraperitoneally injected with D-glucose (1.5 g/kg) after an overnight fast of 16 h with free access to water. Blood glucose levels were measured from the tail vein using a glucometer and strips (ACCU-CHEK, Roche) at 0, 15, 30, 60, and 120 min after glucose injection as reported 10. The insulin tolerance test was performed by measuring blood glucose levels after 6 h of fasting, followed by intraperitoneal injection of 0.5 U/kg insulin (Novolin R) at 0, 15, 30, 60, and 120 min.

### Cell viability assay

Cell viability was assessed using an MTT (3-[4,5-dimethylthiazol-2-yl]-2,5-diphenyltetrazolium bromide, Sigma M2128) assay.

### Analysis of lactate dehydrogenase (LDH) release

LDH levels (an indicator of cell injury) were measured using the LDH Cytotoxicity Detection kit (Takara, MK401) according to the manufacturer's protocol.

### Echocardiography

Transthoracic echocardiography was performed on mice using a Vevo 2100/3100 (VisualSonics).

### Myo-sEV and serum sEV purification and identification

sEVs were isolated from the culture media of cardiomyocytes by ultracentrifugation as the previously described method 19.

### Myo-sEV treatment, labeling, and absorption assay

We employed two methods to assess the impact of *in vivo* treatment with Myo-sEV in mice. The first approach involved utilizing sEVs (1~5×10^10^) secreted by an equivalent number of cardiomyocytes (10^6^, with different treatments). The second approach involved utilizing an equivalent quantity of sEVs (1.2×10^10^). Additionally, we treated the cells with sEVs (1~5×10^9^) secreted by an equivalent number of cardiomyocytes (10^5^, with different treatments) to evaluate their effects *in vitro*.

### Western blotting

Protein was separated by 10%-12% SDS‑PAGE and transferred onto a nitrocellulose membrane (EMD Millipore, USA). The membranes were incubated with the primary antibodies shown in Table S2.

### Transmission electron microscopy

The morphology of sEVs was examined using a transmission electron microscope (TEM, Hitachi H-7650) to observe their ultrastructure, according to established procedures 20.

### miRNA library preparation, microarray chip assay, and data analysis

sEVs for the miRNA microarray chip assay obtained from 50 mL culture medium of C57BL/6 mouse primary cardiomyocytes (from 5 mice) were collected as a single sample and frozen at -80°C as one sample. sEV miRNAs were extracted by using the miRNA isolation Kit (AM1561, Thermo Fisher Scientific, USA) according to the manufacturer's protocol. MicroRNA array analysis was conducted by Oebiotech Co., Ltd. (Shanghai, China).

### Transfection of miRNA mimics and inhibitors into fibroblasts or delivery of miRNA agomiR by tail vein injection

The miR-194-3p mimic, miR-194-3p inhibitors, and their respective negative controls (NC) (Gene Pharma, China) were mixed with TransMessenger Transfection Reagent (Qiagen, 301525) and administered to fibroblasts for 4 h following the manufacturer's protocol. For the *in vivo* experiment, a mixture of 10 nM agomiR-194-3p (Ribibio, China, miR40017148-4-5) and its negative controls (NC) was prepared in 50 µL PBS buffer. This mixture was then administered *via* tail vein injection, with two injections spaced one week apart.

### Reporter gene assay

The HEK293 cells treated with 10 pM miR-194-3p mimic or NC mimic were transfected with 0.4 µg or 0.2 µg of the respective reporter plasmids or the corresponding empty vector (pGL3-Promoter) as a control, along with 20 ng of pRL-SV40 as an internal control reporter. After 48 h post-transfection, the activities of firefly and renilla luciferases were detected using the Dual-Glo^TM^ Luciferase Assay System (Promega, E2920) following the manufacturer's instructions.

### Isolation of miRNAs and total RNAs, and detection of miRNAs, mRNAs, and pri-miRNAs

The sEV-containing small RNAs were extracted using the Total Exosome RNA & Protein Isolation Kit (AM1561, Thermo Fisher Scientific, USA) or the exoRNeasy Serum/Plasma Starter Kit (Qiagen, 77023, USA) following the manufacturer's protocol. miRNAs were detected by the TaqMan™ MicroRNA Assay (Thermo Fisher, 4427975) using the TaqMan MicroRNA Reverse Transcription Kit (Thermo Fisher, 4366596) and the TaqMan Fast Advanced Master Mix (Thermo Fisher,4444557). Total RNA from tissues and cells was isolated using the TRIzol reagent (Invitrogen, 15596018) according to the manufacturer's protocol. The pri-miRNAs were detected using the Pri-miRNA Assay (GenePharma, E22001, China). Gene transcripts, including pri-miRs and predicted target genes, were detected using RevertAid First Strand cDNA Synthesis Kit (ThermoFisher, K1622) and PowerUp™ SYBR^®^ Green Master Mix (ThermoFisher, A25742) on a QuantStudio Real-Time PCR System (ABI,7500) in a 96-well format, respectively. The primer details are available in the online supplement (Table S3).

### Histology and fluorescent immunostaining

Hematoxylin and eosin (H&E) staining was performed on the sections of mouse cardiac tissue. Picrosirius red staining as the fibrosis staining was performed using the Picrosirius Red Stain Kit (Polysciences, Inc. Warrington, PA). Immunostaining was carried out with an anti-α-SMA antibody (Abcam, ab7817).

### Statistical analyses

Statistical data are presented as mean ± SEM (standard error of the mean). GraphPad Prism version 8.0 and SAS (Statistical Analysis System) were used for all statistical analyses except for miRNA chip data. Unpaired two-tailed Student's t-test was used to compare two groups; one-way analysis of variance (ANOVA) followed by Tukey's multiple comparison test was used to compare differences among > 2 groups. All of the statistical experiments were repeated at least three times independently. P < 0.05 indicates a statistical difference between groups.

## Results

### The effect of sEVs released from normal primary cardiomyocytes or high glucose/high lipid (HG/HL)-treated cardiomyocytes (Myo-sEVs^HG/HL^) on fibroblast-to-myofibroblast conversion

**Figure 1:**
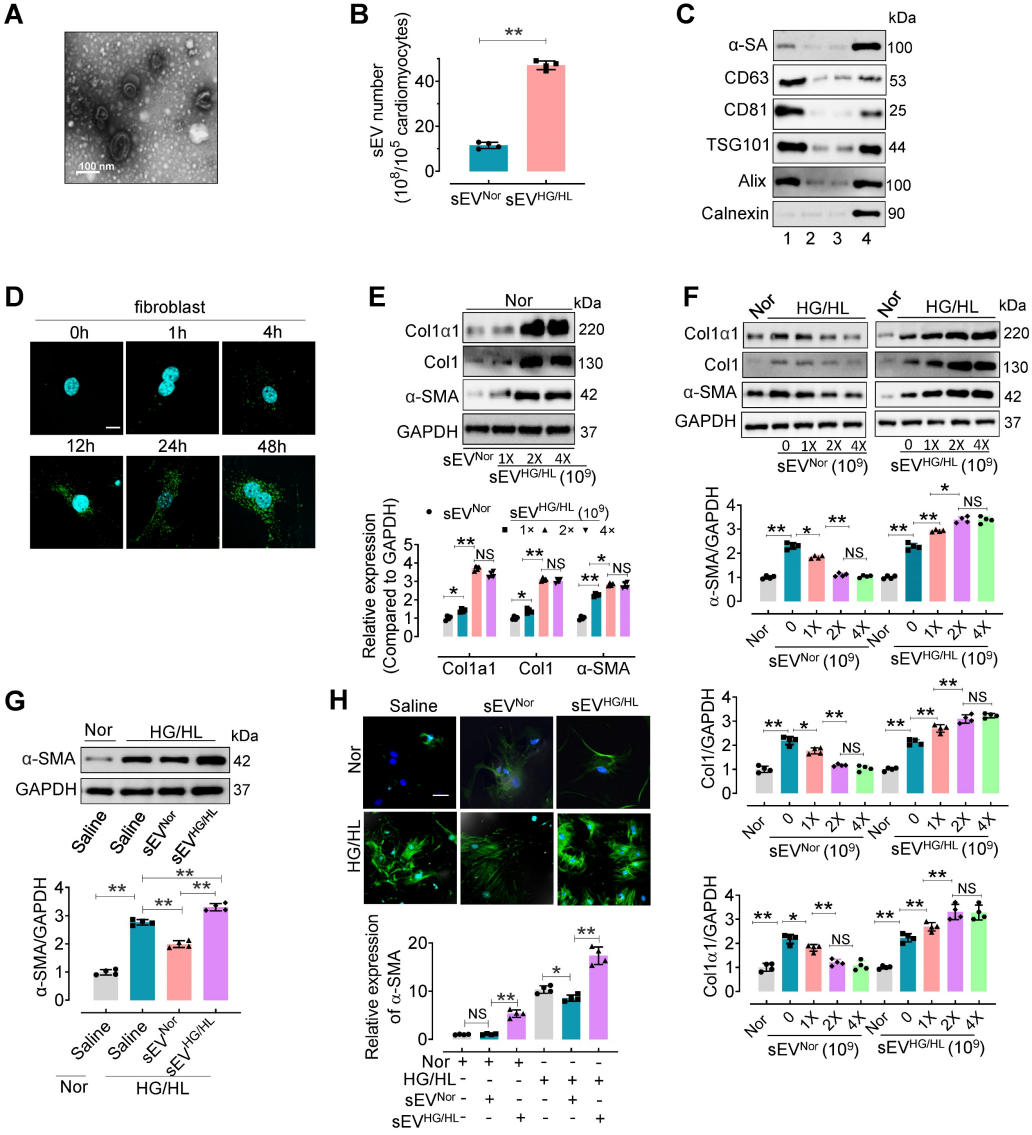
** Myo-sEVs^Nor^ inhibit the fibroblast-to-myofibroblast conversion induced by HG/HL, whereas Myo-sEVs^HG/HL^ exacerbate it.** (**A**) The Myo-sEV morphology was characterized by transmission electron microscopy (TEM). Scale bars: 100 nm. (**B**) Nanosight tracking analysis showed that the number of myo-sEVs^HG/HL^ from HG/HL (25 mM glucose plus 250 μM palmitate)-treated primary cardiomyocytes (from one mouse heart, about 10^5^) was significantly higher than that of myo-sEVs^Nor^. (n = 4). (**C**) Fractions of cardiomyocyte lysate, Myo-sEV as well as cell culture medium with or without Myo-sEV were subjected to Western blotting using cardiomyocyte-specific antibody against α-sarcomeric actin (α-SA), sEV marker antibodies against CD63, CD81; ESCRT protein antibodies against TSG101 and Alix; and an endoplasmic reticulum protein antibody against Calnexin. Lane 1, sEVs derived from primary cardiomyocytes; Lane 2, cardiomyocyte culture medium without Myo-sEVs; Lane 3, cardiomyocyte culture medium with Myo-sEVs; Lane 4, cardiomyocyte lysate. (**D**) PKH67-labeled Myo-sEVs were taken up by cardiac fibroblasts in a time-dependent manner. Scale bars: 5 μm. (**E**) The translational expression levels of Collagen1α1, Collagen1, and α-SMA were examined after different Myo-sEV^HG/HL^ gradient dosage treatments (1×10^9^, 2×10^9^, and 4×10^9^) with Myo-sEV^Nor^ (1×10^9^) as the control in normal treatment (n = 4). (**F**) Fibroblasts were treated with normal glucose/normal lipid (Nor) or HG/HL for 24 h, followed by different gradient doses of Myo-sEV^Nor^ and Myo-sEV^HG/HL^ for another 24 h. The protein expression levels of Collagen1α1, Collagen1, and α-SMA were examined by Western blotting (n = 4). (**G, H**) Fibroblasts were treated with normal glucose/normal lipid (Nor) or HG/HL for 24 h, followed by the treatment with either Myo-sEVs^Nor^ or Myo-sEVs^HG/HL^ from an equal number of cardiomyocytes (10^5^) for another 24 h. Then the translational expression of α-SMA was detected by Western blotting (G) or immunostaining (green) (H). (n = 4). Scale bars: 20 μm. All values are presented as mean ± SEM. P values were calculated by unpaired two-tailed Student's t-test (B) or one-way ANOVA followed by Tukey's test (E-H). *p < 0.05, **p <0.01.

To investigate the influence of Myo-sEVs on cardiac fibroblast-to-myofibroblast conversion, we first isolated Myo-sEVs by ultracentrifugation. Before this, we evaluated the viability and purity of primary cardiomyocytes treated with normal glucose/normal lipid (NG/NL) or high glucose/high lipid (HG/HL, consisting 25 mM glucose and 250 μM palmitate) for 24 h (Figure S1A-C). The purity of cardiomyocytes was 95.68% ± 2.58 (Figure S1A). No significant difference was observed between primary cardiomyocytes treated with NG/NL or HG/HL in terms of cell viability (MTT assay, Figure S1B) and cell survival (LDH release assay, Figure S1C). Then the purity of Myo-sEVs was measured. Electron microscopy showed that most Myo-sEVs exhibited classic disc-shaped vesicles with an average diameter of around 100 nm (Figure 1A). Nanoparticle tracking analysis revealed Myo-sEVs with an average size of 114.0 ± 43.2 nm (Figure S1D), and the amount of Myo-sEVs^HG/HL^ was approximately 4-fold that of Myo-sEVs^Nor^ (Figure 1B). The Myo-sEV fraction was enriched in sEV-associated proteins such as CD63, CD81, ALIX, and TSG101, whereas the endoplasmic reticulum protein Calnexin was barely detectable. Additionally, the cardiomyocyte protein, α-sarcomeric actin (α-SA), was observed in the Myo-sEV fraction (Figure 1C). Fluorescence staining results confirmed the uptake of PKH67-labeled Myo-sEVs by cardiac fibroblasts *in vitro* in a time-dependent manner (Figure 1D) or by C57BL/6 mouse heart cells *in vivo*, whether the mice were administered through intramyocardial injection or tail vein injection (Figure S1E and F). Following the confirmation of the high purity (94.32% ± 3.31) and unchanged viability of cardiac fibroblasts treated with Myo-sEVs^HG/HL^ (Figure S1G-I), the transcriptional and translational levels of Collagen1α1, Collagen1, and α-SMA were subsequently detected in these fibroblasts with Myo-sEV^Nor^ (1×10^9^, as the control) or different Myo-sEV^HG/HL^ gradient doses (1×10^9^, 2×10^9^, and 4×10^9^) treatment. The results consistently showed that all three molecules were upregulated by sEV^HG/HL^ in a dose-dependent manner (Figure 1E and Figure S1J). Furthermore, the protein levels of Collagen1α1, Collagen1, and α-SMA were measured in fibroblasts treated with HG/HL or 24 h and gradient doses (0, 1×10^9^, 2×10^9^, and 4×10^9^) of Myo-sEVs^Nor^ or Myo-sEV^HG/HL^ for another 24 h. The results revealed that Myo-sEVs^Nor^ mitigated the expression of Collagen1α1, Collagen1, and α-SMA in a dose-dependent manner, whereas Myo-sEV^HG/HL^ exacerbated their expression (Figure 1F). Subsequently, we treated fibroblasts with a dose of 2×10^9^ Myo-sEVs^Nor^ or 2×10^9^ Myo-sEV^HG/HL^ which had been shown to have a significant effect on the fibrotic response according to the result of Figure 1F. Consistent with the former result, we observed that treatment with Myo-sEVs^Nor^ reduced the expression of α-smooth muscle actin (α-SMA) induced by HG/HL. In contrast, Myo-sEVs^HG/HL^ significantly augmented α-SMA expression in fibroblasts (Figure S1K-M). These findings suggest that an equivalent number of Myo-sEVs^Nor^ and Myo-sEVs^HG/HL^ had opposite effects on the fibrotic response, with Myo-sEVs^Nor^ ameliorating HG/HL-induced fibroblast-to-myofibroblast conversion and Myo-sEVs^HG/HL^ exacerbating it. Finally, the same measurements were carried out in fibroblasts treated with either Myo-sEVs^Nor^ or Myo-sEVs^HG/HL^ (doses ranging from 1 ~ 5×10^9^, diluted in 2 mL of culture medium), derived from an equal number of cardiomyocytes (10^5^), and the similar results were obtained that Myo-sEVs^Nor^ treatment effectively reduced the expression of α-smooth muscle actin (α-SMA) induced by HG/HL. Conversely, Myo-sEVs^HG/HL^ treatment led to an increase in α-SMA expression in fibroblasts (Figure 1G-H, and Figure S1N).

### Myo-sEVs^Nor^ protect against cardiac fibrosis in mice treated with HFD plus STZ, whereas Myo-sEVs^HG/HL^ exacerbate cardiac fibrosis in these mice

**Figure 2:**
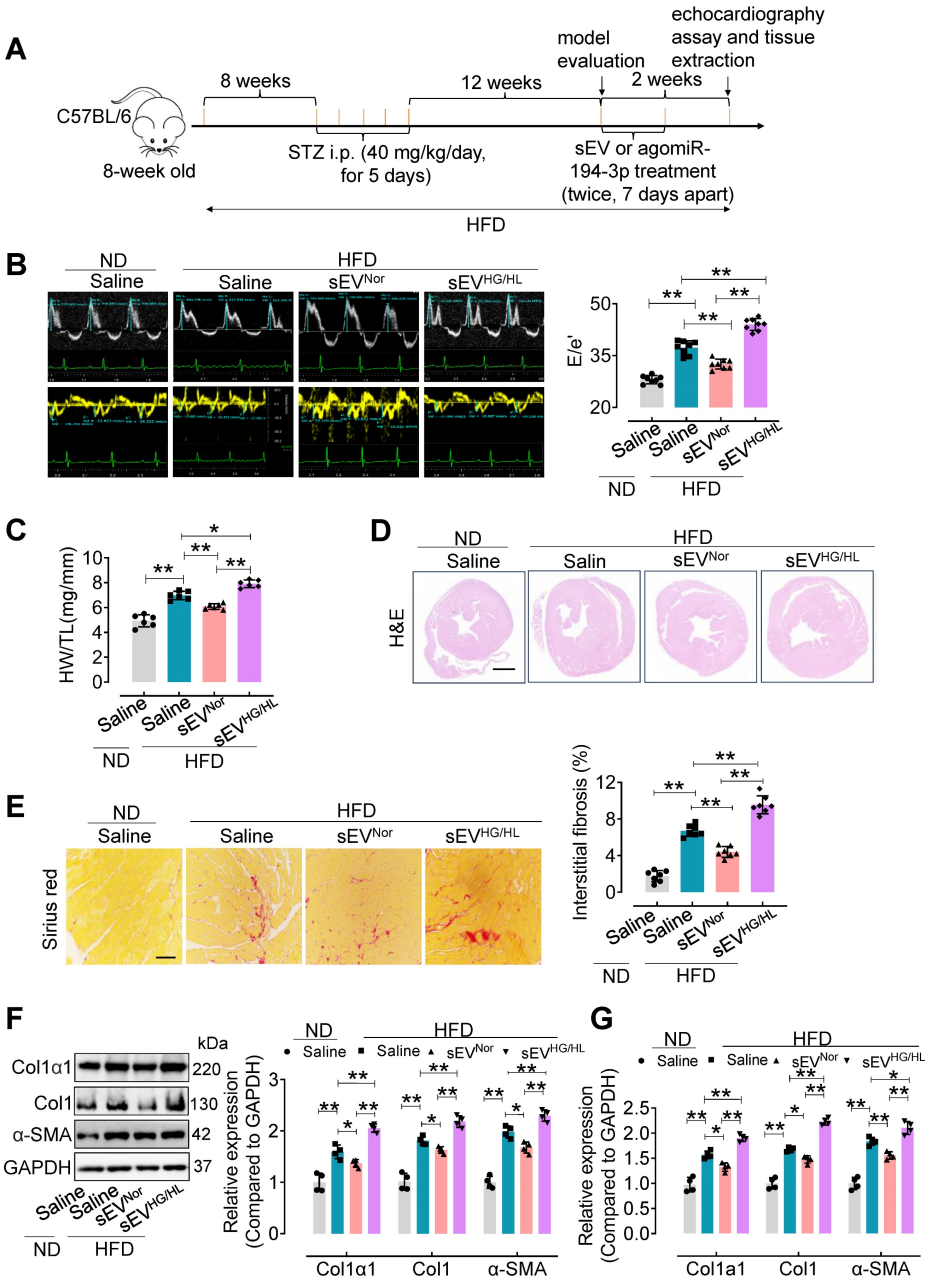
** The effect of Myo-sEVs from an equal number of cardiomyocytes on cardiac fibrosis in HFD plus STZ-induced diabetic mice.** (**A**) A schematic representation of the construction process of HFD-induced type 2 diabetes mellitus mouse model and the treatment with Myo-sEV or agomiR-194-3p. Type 2 diabetes mellitus mouse model induced by a high-fat diet (HFD, about 23 weeks) combined with trace intraperitoneal injection of STZ (i.p. 40 mg/kg/day, for 5 days). After model evaluation, Myo-sEVs^Nor^, Myo-sEVs^HG/HL^, or agomiR-194-3p were administered *via* tail vein injections twice at 7-day intervals. (**B**) E-waves (upper panel) were measured using pulsed wave (PW) Doppler from a 4-chamber view of the lateral mitral valve. Early diastolic (e') velocities were obtained from the Tissue Doppler signal of the mitral annulus (lower panel). The ratio of peak E to peak e' significantly increased after treatment with Myo-sEVs^HG/HL^. (n = 6). (**C**) The ratio of heart weight to tibia length (HW/TL) in HFD mice increased when treated with Myo-sEVs^HG/HL^ compared to Myo-sEVs^Nor^. (n = 6). (**D**) Hematoxylin and eosin staining of whole mouse heart histological sections were performed after normal diet (ND) or HFD mice were treated* in vivo* with Myo-sEV^Nor^ and Myo-sEV^HG/HL^. Scale bar: 1 mm. (**E**) Representative images of histological sections and quantification of Picrosirius red-stained areas showing collagen deposition in the hearts of ND or HFD mice *in vivo* treated with Myo-sEV^Nor^ and Myo-sEV^HG/HL^. (n = 7). Scale bars: 100 μm. (**F, G**) The protein (F) and mRNA (G) expression levels of Col1α1, Col1, and α-SMA were assessed by Western blotting and qPCR separately in the hearts of mice subjected to the indicated treatment. (n = 4). All values are presented as mean ± SEM. P values were calculated by one-way ANOVA followed by Tukey's test. *p < 0.05, **p < 0.01.

Furthermore, an *in vivo* study was conducted to assess the impact of Myo-sEVs on diabetic cardiac fibrosis in high-fat diet (HFD)-induced type 2 diabetes. The model was induced by a combination of HFD and moderate doses of streptozotocin (STZ) (Figure 2A) 21 and validated according to the intraperitoneal glucose tolerance test (IP GTT) and intraperitoneal insulin tolerance test (IP ITT) (Figure S2A and B). Physiological and biochemical parameters were also assessed in the HFD diabetic mice (Figure S2C-F). Then, cardiac function and cardiac fibrosis were evaluated *in vivo* following the administration of Myo-sEVs from the same number of cardiomyocytes (10^6^). The results showed that tail vein injection of Myo-sEVs^HG/HL^ exacerbated diastolic dysfunction in left ventricular (E/e') in the HFD mice, while Myo-sEVs^Nor^ improved it (Figure 2B, the different scales as indicated). Additionally, treatment with HFD led to an increase in the heart weight to tibia length (HW/TL) ratio, which was significantly reversed by Myo-sEVs^Nor^, whereas Myo-sEVs^HG/HL^ enhanced the HW/TL ratio induced by HFD (Figure 2C). The representative images of hematoxylin and eosin staining demonstrated the relative size of the cross-sectional view in the same position of the hearts after the mouse with the indicated treatment (Figure 2D). Myo-sEVs^HG/HL^ administration exacerbated the size of cardiac fibrosis compared to HFD treatment alone, whereas Myo-sEVs^Nor^ reduced HFD-induced cardiac fibrosis (Figure 2E). Consistent with these findings, the expression of Col1α1, Col1, and α-SMA in the heart tissue of HDF mice was enhanced after Myo-sEVs^HG/HL^ treatment, whereas Myo-sEVs^Nor^ reduced it, as shown by the results of Western blotting and qPCR analysis (Figure 2F and G). Similar results were observed in the HFD mice treated with the same number of Myo-sEVs^Nor^ and Myo-sEVs^HG/HL^ (1.2×10^10^), where Myo-sEVs^HG/HL^ exacerbated diastolic dysfunction and cardiac fibrosis, increased the HW/BW ratio and induced the expression of Col1α1, Col1, and α-SMA, whereas Myo-sEVs^Nor^ restored these indicators in the heart tissue of HFD mice (Figure S3). However, except for echocardiographic parameters (Table S4), no significant differences were found in other physiological and biochemical parameters in HFD mice, regardless of the treatment with Myo-sEVs^Nor^ or Myo-sEVs^HG/HL^ (Figure S4 and Figure S5).

Although we observed significant differences in Myo-sEVs^Nor^ or Myo-sEVs^HG/HL^ *in vivo*-treated diabetic mice, there were no variations in cardiac function or cardiac fibrosis in Myo-sEVs^Nor^ or Myo-sEVs^HG/HL^* in vivo*-treated non-diabetic mice (Figure S6A-E), apart from an increase in Col1α1, Col1, and α-SMA expression in cardiac tissue after Myo-sEVs^HG/HL^ administration (Figure S6F and G).

### The effects of sEVs from primary cardiomyocytes of control mice (Myo-sEVs^Con^) and *db/db* mice (Myo-sEVs*^db/db^*) on cardiac fibrosis in diabetic mice

**Figure 3:**
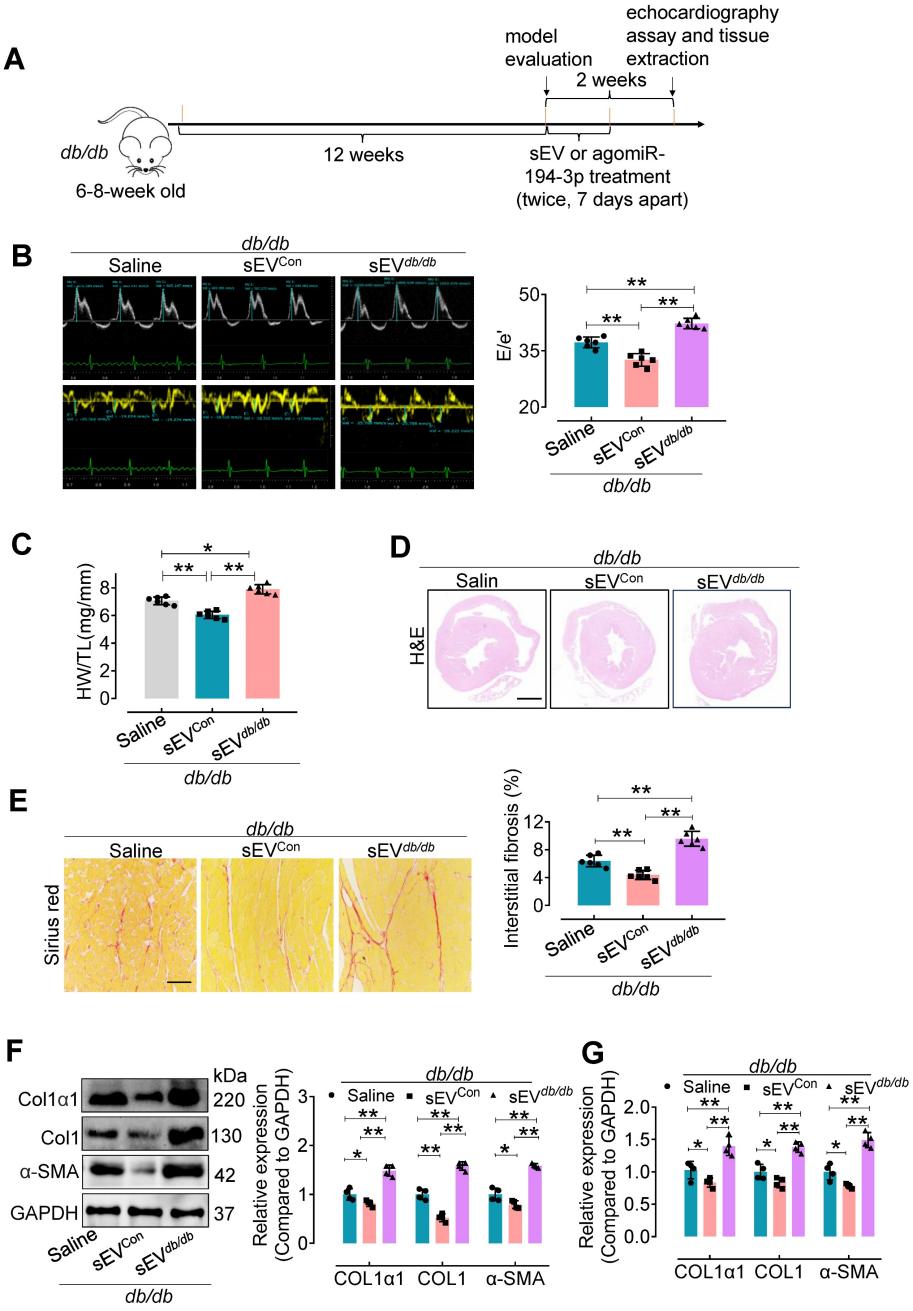
** Myo-sEVs*^db/db^* exacerbate cardiac fibrosis in* db/db* mice.** (**A**) A schematic representation of the *db/db* type 2 diabetes mellitus mouse model and the course of Myo-sEV or agomiR-194-3p treatment. (**B**) An increase in the E to e' peak ratio was observed in the hearts of* db/db* mice after *in vivo* treatment with Myo-SEV*^db/db^*. (n = 6). (**C**) An increased HW/TL was observed in *db/db* mice after treatment with Myo-SEV*^db/db^* compared to those treated with Myo-sEV^Con^. (n = 6). (**D**) Hematoxylin and eosin staining of whole mouse heart histological sections were performed after *db/db* mice were treated* in vivo* with Myo-sEV^Con^ and Myo-sEV*^db/db^*. Scale bars: 1 mm. (**E**) Picrosirius red-staining showing collagen deposition in mouse hearts after the *db/db* mice were treated* in vivo* with Myo-sEV^Con^ and Myo-sEV*^db/db^*. Scale bars: 100 μm. (n = 6). (**F, G**) The protein (F) and mRNA (G) expression levels of Col1α1, Col1, and α-SMA were separately quantified using Western blotting and qPCR in the hearts of mice subjected to the indicated treatment. (n = 4). All values are presented as mean ± SEM. P values were calculated by one-way ANOVA followed by Tukey's test. *p < 0.05, **p < 0.01.

We also used a genetic model of type 2 diabetes to confirm the impact of Myo-sEVs on cardiac fibrosis (Figure 3A). Similar to the effect of Myo-sEVs^Nor^ on cardiac fibrosis in HFD mice, our results showed that Myo-sEVs^Con^ significantly improved diastolic dysfunction (Figure 3B), attenuated relative heart weight and diabetic cardiac fibrosis in *db/db* mice (Figure 3C-E).

Additionally, they reduced the expression of Col1α1, Col1, and α-SMA in the hearts of *db/db* mice (Figure 3F and G). On the contrary, Myo-sEVs*^db/db^* exacerbated diastolic dysfunction and diabetic cardiac fibrosis (Figure 3B-E), together with increased the expression of Col1α1, Col1, and α-SMA in the hearts of *db/db* mice (Figure 3F and G). However, except for echocardiographic parameters, no significant differences were observed in other physiological and biochemical parameters in *db/db* mice, regardless of whether the mice were treated with Myo-sEVs^Con^ or Myo-sEVs*^db/db^* (Figure S7).

### miR-194-3p is reduced in Myo-sEVs^HG/HL^ or Myo-sEVs*^db/db^*

**Figure 4:**
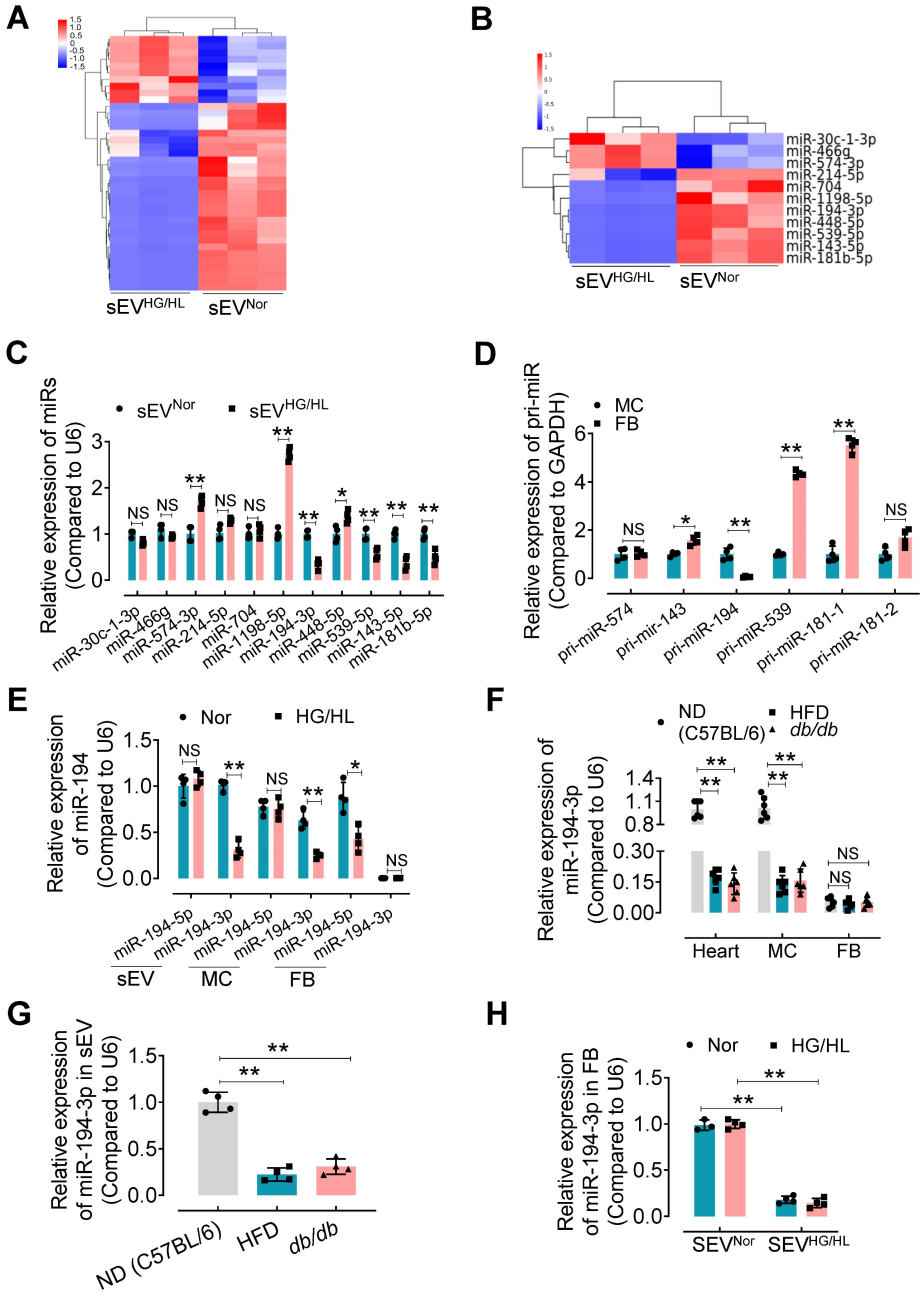
** miR-194-3p is reduced in Myo-sEVs^HG/HL^.** (**A**) Different miRNA levels were identified by a microRNA array. 10 up-regulated miRNAs and 28 down-regulated miRNAs were identified in Myo-sEVs^HG/HL^ compared to Myo-sEVs^Nor^. (**B**) 11 different miRNAs with identical sequences in human and mouse were selected. (filtering criteria, p < 0.05, fold change > 2.0, n = 3). (**C**) The relative levels of mature miRNAs in Myo-sEV^Nor^ or Myo-sEV^HG/HL^ were quantified using qPCR. (n = 4). (**D**) The relative expression of pri-miRs was detected by qPCR in primary cardiomyocytes and fibroblasts. (n = 4). (**E**) qPCR results showed the relative levels of the mature miR-194 family (miR-194-3p and miR-194-5p) in Myo-sEVs, cardiomyocytes, and fibroblasts under Nor or HG/HL treatment. (n = 4). (**F**) The relative miR-194-3p levels were measured in the hearts, primary cardiomyocytes, and fibroblasts obtained from C57BL/6 mice on a normal diet (ND), high-fat diet (HFD), and *db/db* mice. (n = 6). (**G**) The relative miR-194-3p levels in Myo-sEVs derived from cardiomyocytes from ND, HFD, and *db/db* mice. (n = 4). (**H**) The relative expression of miR-194-3p was measured in primary cardiac fibroblasts treated with Nor or HG/HL in the presence of Myo-sEV^Nor^ or Myo-sEV^HG/HL^. (n = 4). All values are presented as mean ± SEM. P values were calculated by unpaired two-tailed Student's t-test (C-E) or one-way ANOVA followed by Tukey's test (F-H). *p < 0.05, **p < 0.01.

sEV miRNAs play a key role in the regulation of recipient cell function, particularly in the heart 10. Therefore, we focused on identifying specific miRNAs in Myo-sEVs that may contribute to cardiac fibroblast-to-myofibroblast conversion under diabetic conditions. Using unbiased microRNA array analysis, we identified 10 up-regulated miRNAs and 28 down-regulated miRNAs in Myo-sEVs^HG/HL^ compared to Myo-sEVs^Nor^ (filtering criteria, p < 0.05, fold change >2.0. Figure 4A, Figure S8A, and Table S5). From the 38 miRNAs, we selected 11 miRNAs with identical sequences in humans and mice for Gene ontology analysis using the DAVID software (version 7.2) (Figure 4B and Figure S8B). Furthermore, we set two criteria to screen the target miRNA from the 11 candidates: confirmation through qPCR assay, and enrichment in cardiomyocytes rather than cardiac fibroblasts. Based on these criteria, 5 miRNAs in Myo-sEVs from different sources were validated by qPCR including miR-574-3p, miR-143-5p, miR-194-3p, miR-539-5p, and miR-181b-5p, with the same trends as those seen in the microRNA array results (Figure 4C). Then we detected the corresponding pri-miRs in sEV-free medium-cultured cardiomyocytes and fibroblasts. Of note, the expression of pri-miR-194 was obvious in cardiomyocytes but scarce in cardiac fibroblasts (Figure 4D). This suggests that miR-194-3p is endogenously expressed in cardiomyocytes but not in cardiac fibroblasts. Importantly, we found that Myo-sEVs contain a higher level of miR-194-3p compared to its cellular source under normal conditions, indicating that Myo-sEVs are the pivotal means for cardiomyocyte miR-194-3p to exert its function (Figure 4E). Meanwhile, there was a notable reduction in the levels of miR-194-3p in the hearts of HFD mice or *db/db* mice as well as in primary cardiomyocytes isolated from the two diabetic mouse models (Figure 4F) or treated with HG/HL (Figure 4E). This decline in miR-194-3p levels was also observed in Myo-sEVs^HFD^ and Myo-sEVs*^db/db^* (Figure 4G). However, the levels of miR-194-3p in primary cardiac fibroblasts were nearly undetectable, regardless of HG/HL treatment (Figure 4E) or origin from HFD mice or *db/db* mice (Figure 4F). Nevertheless, upon the treatment with Myo-sEVs^Nor^ (24 h), miR-194-3p became detectable in cardiac fibroblasts and reached significantly higher levels compared to the Myo-sEVs^HG/HL^-treated group, irrespective of normal or HG/HL conditions (Figure 4H). These results indicate that fibroblasts have naturally low levels of endogenous miR-194-3p and that Myo-sEVs play a crucial role in facilitating the transport of miR-194-3p from cardiomyocytes to cardiac fibroblasts. In addition to the heart, the relative expression of miR-194-3p was measured in several other tissues, including the lung, kidney, liver, aorta, white fat, and brown fat, in normal diet (ND), a high-fat diet (HFD), and *db/db* mice. Notably, among all the tissues, miR-194-3p expression was significantly reduced only in the heart of diabetic and HFD mice, with a slightly inverse trend in the aorta (Figure S8C).

### agomiR-194-3p mitigates diabetic cardiac fibrosis in HFD and *db/db* mice

**Figure 5:**
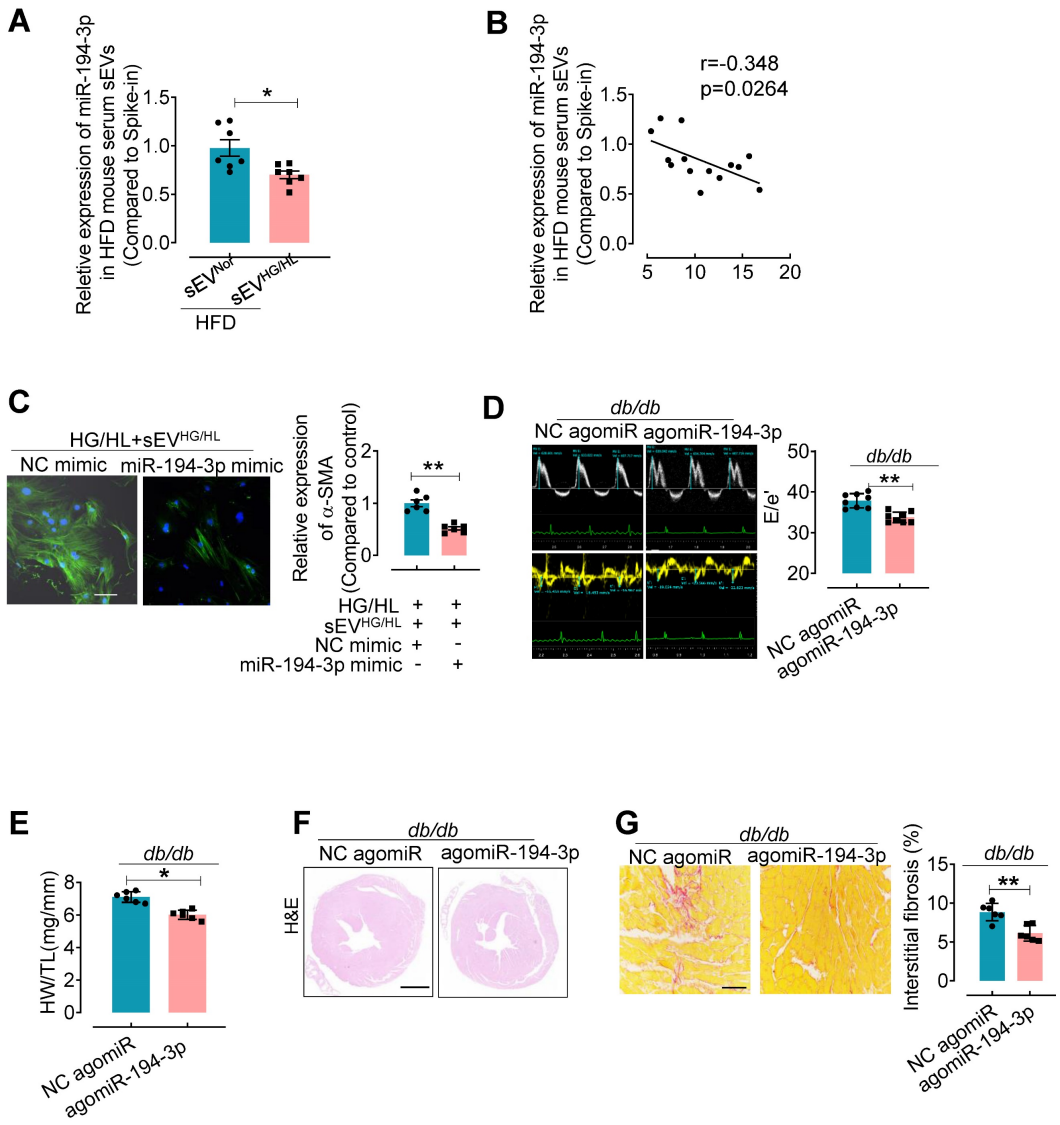
** agomiR-194-3p inhibits cardiac fibroblast-to-myofibroblast conversion and ameliorates diabetic cardiac fibrosis in *db/db* mice.** (**A**) qPCR assay showed the relative miR-194-3p levels in serum sEVs of mice treated with Myo-sEV^Nor^ or Myo-sEV^HG/HL^, both derived from an equal number of cardiomyocytes. (cel-miR-39 miRNA as a spike-in control, n = 7). (**B**) The levels of miR-194-3p in serum sEVs exhibited a negative correlation with the degree of cardiac fibrosis in Myo-sEVs^HG/HL^-treated HFD mice, as determined through Spearman's correlation analysis. (n = 14). (**C**) Immunostaining for α-SMA (green) in fibroblasts treated with HG/HL plus Myo-sEV^HG/HL^ with or without miR-194-3p mimic. Scale bars: 20 μm. (n = 6). (**D**) The E-waves (upper panel) were measured using pulsed wave (PW) Doppler from a 4-chamber view of the lateral mitral valve. Early diastolic (e') velocities were obtained from the Tissue Doppler signal of the mitral annulus (lower panel). The ratio of peak E to peak e' showed a significant decrease in *db/db* mice after treatment with agomiR-194-3p. (n = 8). (**E**) The HW/TL of *db/db* mice showed a decrease after treatment with agomiR-194-3p compared to the negative control (NC agomiR). (n = 6). (**F**) Hematoxylin and eosin staining was performed on histological sections of whole mouse hearts from *db/db* mice treated in *vivo* with agomiR-194-3p and NC agomiR. Scale bar: 1 mm. (**G**) Picrosirius red-staining showing collagen deposition in mouse hearts after *in vivo* treatment of *db/db* mice with agomiR-194-3p and NC agomiR. Scale bars: 100 μm. (n = 6). All values are presented as mean ± SEM. P values were calculated by unpaired two-tailed Student's t-test. *p < 0.05, **p < 0.01.

To further elucidate the relationship between sEV miR-194-3p and diabetic cardiac fibrosis* in vivo*, we examined the levels of miR-194-3p in serum sEVs and their correlation with cardiac fibrosis in HFD mice treated with Myo-sEVs^HG/HL^. Results revealed that significantly lower levels of miR-194-3p in serum sEVs of HFD mice receiving either the Myo-sEVs^HG/HL^ from the same number of cardiomyocytes (10^6^, Figure 5A) or an equal amount of Myo-sEVs^HG/HL^ (1.2×10^10^, Figure S9A) as compared with their respective controls. Of note, miR-194-3p levels in serum sEVs negatively correlated with the extent of cardiac fibrosis in Myo-sEVs-treated HFD mice (Figure 5B and Figure S9B). Furthermore, transfection with 194-3p mimic significantly attenuated the fibroblast-to-myofibroblast conversion induced by HG/HL plus Myo-sEVs^HG/HL^ (Figure 5C and Figure S9C). As expected, the overexpression of miR-194-3p with agomiR-194-3p (Figure S9D and E) significantly alleviated cardiac left ventricular diastolic dysfunction (E/e'), reduced HW/TL ratio, and blocked cardiac fibrosis (as seen in picrosirius red staining) in the hearts of *db/db* mice (Figure 5D-G) or HFD mice (Figure S10). However, except for ejection fraction (EF) and fractional shortening (FS), agomiR-194-3p had no impact on other physiological and biochemical indices (Figure S11, Figure S12, and Table S6).

### TGFβR2 is a novel target of miR-194-3p in fibroblasts and is upregulated in response to Myo-sEVs^HG/HL^ or diabetes, which is reversed by overexpression of miR-194-3p

**Figure 6:**
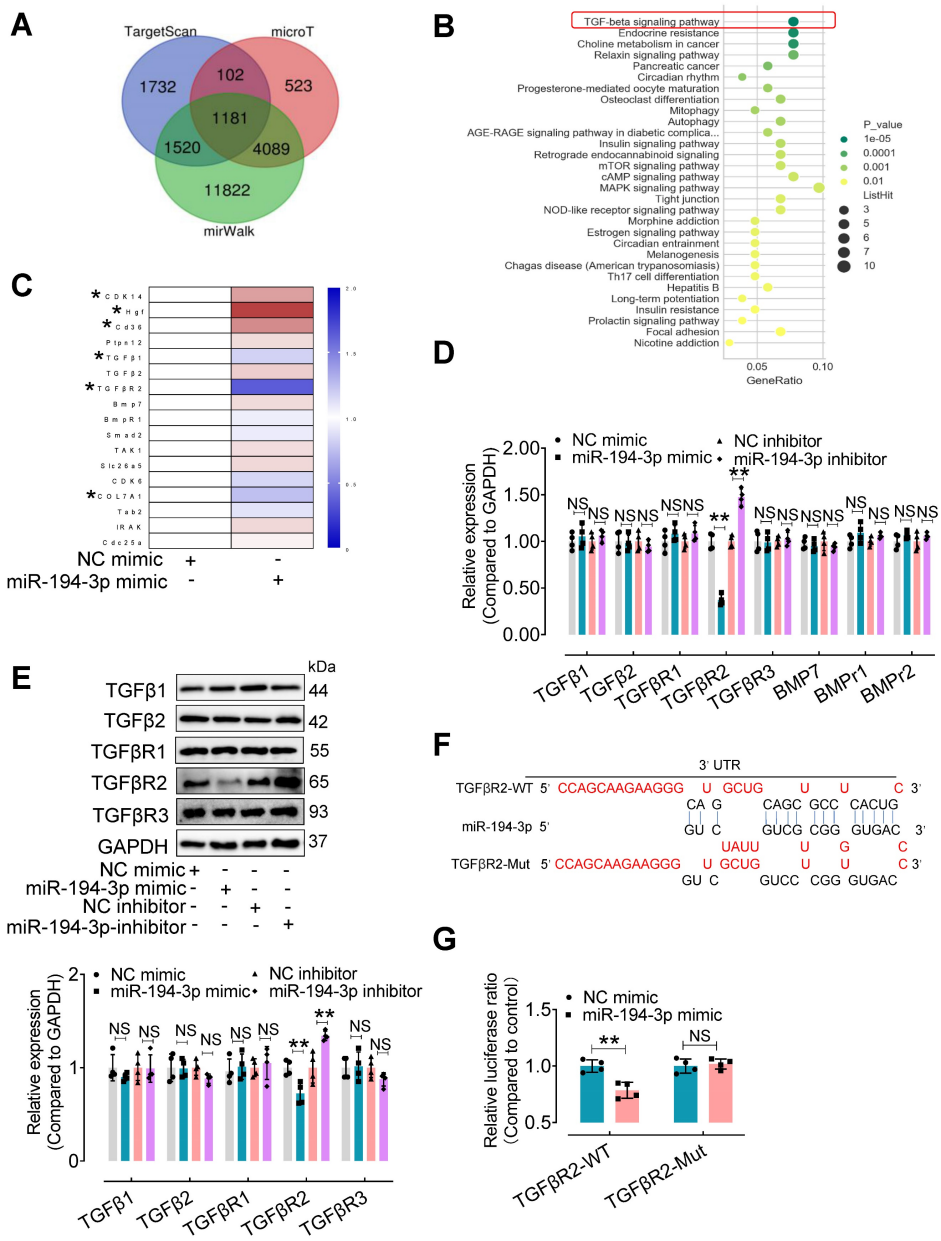
** TGFβR2 expression is regulated by miR-194-3p.** (**A**) Venn diagram showing the number of target genes of miR-194-3p predicted separately by TargetScan, mirWalk, and microT databases. (**B**) KEGG pathways were used to analyze the top enriched functions of the 1,181 predicted identical target genes of miR-194-3p. (**C**) qPCR analysis of the top 17 differentially expressed miR-194-3p target genes in fibroblasts after miR-194-3p mimic treatment. (n = 4). (**D, E**) The relative mRNA (D) and protein (E) expression levels of molecules related to the TGFβ signaling pathway were assessed in fibroblasts after miR-194-3p mimic or miR-194-3p inhibitor treatment. (n = 4). (**F**) The predicted binding sites of miR-194-3p on TGFβR2 3'UTR regions. (**G**) Luciferase reporter gene assay showed that miR-194-3p mimic inhibited the luciferase activity when the luciferase reporter gene was linked to the TGFβR2 3'UTR sequence in the reporter plasmid, while the mutant sequence of the TGFβR2 3'UTR reversed the effect. (n = 4). All values are presented as mean ± SEM. P values were calculated by unpaired two-tailed Student's t-test. *p < 0.05, **p < 0.01.

To explore the mechanism by which miR-194-3p protects against cardiac fibroblast-to-myofibroblast conversion, we first performed bioinformatic analyses. 1,181 mouse genes were predicted as potential targets of miR-194-3p using three different databases (miRWalk, microT, and Targetscan) (Figure 6A). Furthermore, using the DAVID Functional Annotation Bioinformatics Analysis (version 7.2), we identified the TGFβ signaling pathway as the most likely pathway associated with miR-194-3p (Figure 6B). To validate these predictions, we conducted qPCR analyses on fibroblasts treated with either miR-194-3p mimic or negative control (NC) to detect the top 17 predicted target genes (Figure 6C, Table S3). We also evaluated the relative expression of molecules involved in the TGFβ signaling pathway in fibroblasts treated with miR-194-3p mimic or miR-194-3p inhibitor using qPCR and Western blotting. Strikingly, our findings revealed that out of the predicted targets, only TGFβR2 consistently exhibited regulation by miR-194-3p at both the transcriptional and translational levels, with decreased expression in the presence of miR-194-3p mimic, and increased expression after miR-194-3p inhibitor treatment (Figure 6D and E). Targetscan software predicted specific binding sites of miR-194-3p on the 3'UTR regions of TGFβR2 mRNA (Figure 6F). A luciferase reporter assay further confirmed that miR-194-3p downregulated TGFβR2 at the transcription level (Figure 6G). Collectively, these results strongly suggest that TGFβR2 is a novel target gene of miR-194-3p involved in the regulation of cardiac fibroblast-to-myofibroblast conversion.

**Figure 7:**
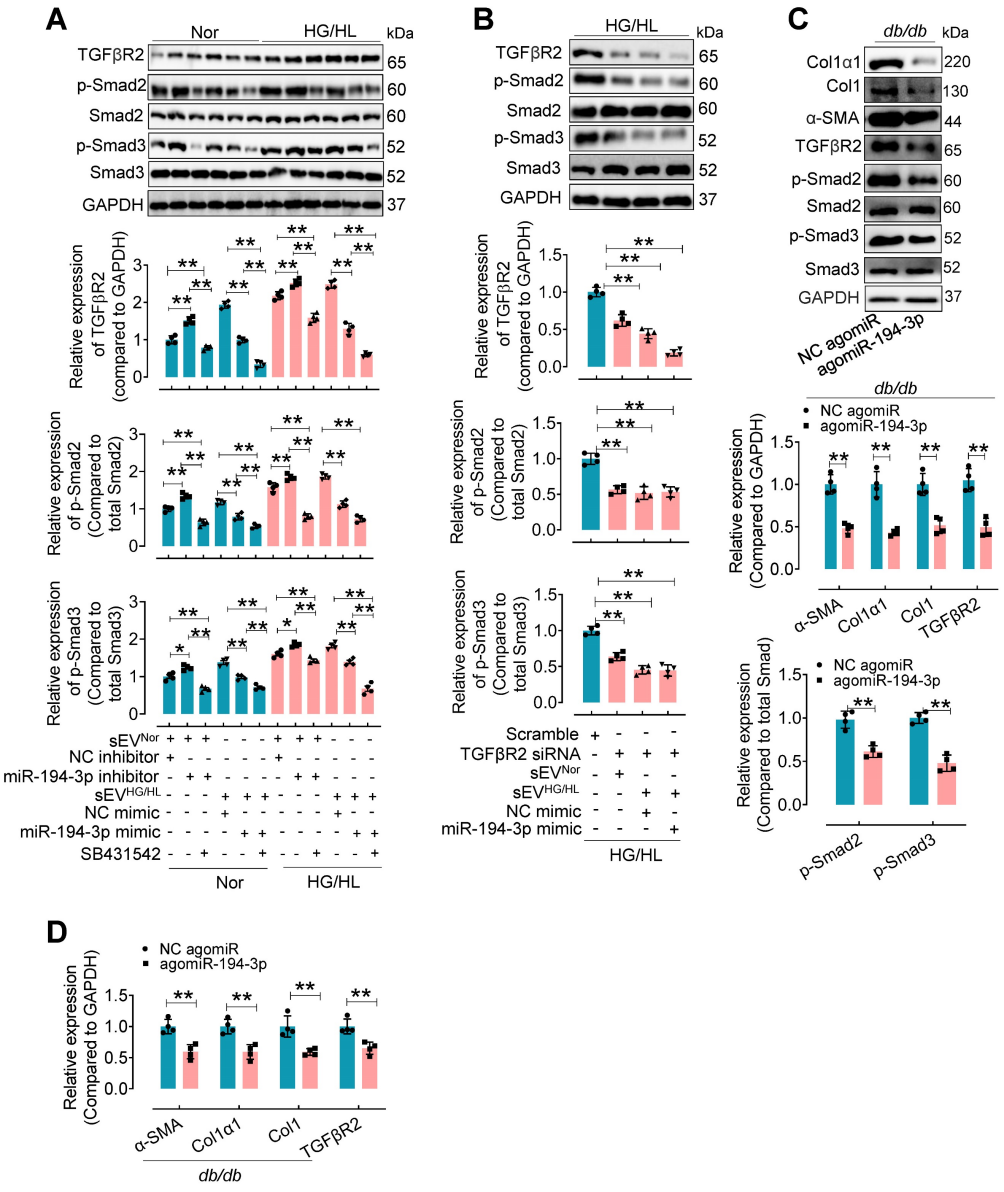
** The upregulated expression of TGFβR2 by Myo-sEVs^HG/HL^ or diabetes was reversed by miR-194-3p.** (**A**) Western blotting analysis of molecules related to the TGFβ signaling pathway was conducted in cardiac fibroblasts after 24-hour treatment with Myo-sEV^ HG/HL^, miR-194-3p mimic or inhibitor, and SB431542 under normal or HG/HL conditions. (n = 4). (**B**) Western blotting analysis of molecules related to the TGFβ signaling pathway was conducted in fibroblasts treated with TGFβR2 siRNA, Myo-sEV^ HG/HL^, and miR-194-3p mimic under HG/HL conditions. (n = 4). (**C-D**) The protein (C) and mRNA (D) expression levels of Col1α1, Col1, α-SMA, and molecules related to the TGFβ signaling pathway were assessed by Western blotting and qPCR in heart tissue from *db/db* mice treated with agomiR-194-3p and a negative control (NC agomiR). (n = 4). All values are presented as mean ± SEM. P values were calculated by unpaired two-tailed Student's t-test (C-D) or one-way ANOVA followed by Tukey's test (A-B). *p < 0.05, **p < 0.01.

To investigate the regulation of TGFβR2 in diabetic cardiac fibrosis and the involvement of miR-194-3p, we conducted additional experiments using cardiac fibroblasts under the treatment of HG/HL, Myo-sEV^HG/HL^, miR-194-3p mimic, miR-194-3p inhibitor, TGFβR2 siRNA, and TGFβR2 inhibitor. Results demonstrated that TGFβR2, p-Smad2, and p-Smad3 were upregulated by miR-194-3p inhibitor, Myo-sEV^HG/HL^, and HG/HL. However, this upregulation was significantly restored by TGFβ inhibitor SB431542 (which did not alter TGFβR2 expression), miR-194-3p mimic, or TGFβR2 siRNA (TGFβR2 siRNA site 874). (Figure 7A and B, and Figure S13A to C). Furthermore, *in vivo* studies with *db/db* mice or HFD mice treated with agomiR-194-3p showed a dramatic decrease in protein and mRNA expression levels of Col1α1, Col1, p-Smad2, p-Smad3, α-SMA, and TGFβR2 in the heart (Figure 7C and D, and Figure S13D and E). TGFβ is well-known for its ability to induce cardiac fibroblast-to-myofibroblast conversion, ultimately resulting in cardiac fibrosis. Here, we also detected the protein levels of the TGFβ-Smad signaling pathway (TGFβR2, p-Smad2, and p-Smad3) and myocardial fibrosis markers (Col1α1, Col1, α-SMA) in fibroblasts after 10 ng/mL TGFβ plus miR-194-3p mimic, miR-194-3p inhibitor and TGFβR2 siRNA treatment. Results demonstrated that the TGFβ-induced expression of these proteins was significantly blocked by miR-194-3p mimic or TGFβR2 siRNA, but upregulated by miR-194-3p inhibitor compared to the negative control (NC). (Figure Figure S14A-B).

### Serum sEVs from type 2 diabetes patients contain lower miR-194-3p and induce cardiac fibroblast-to-myofibroblast conversion

**Figure 8:**
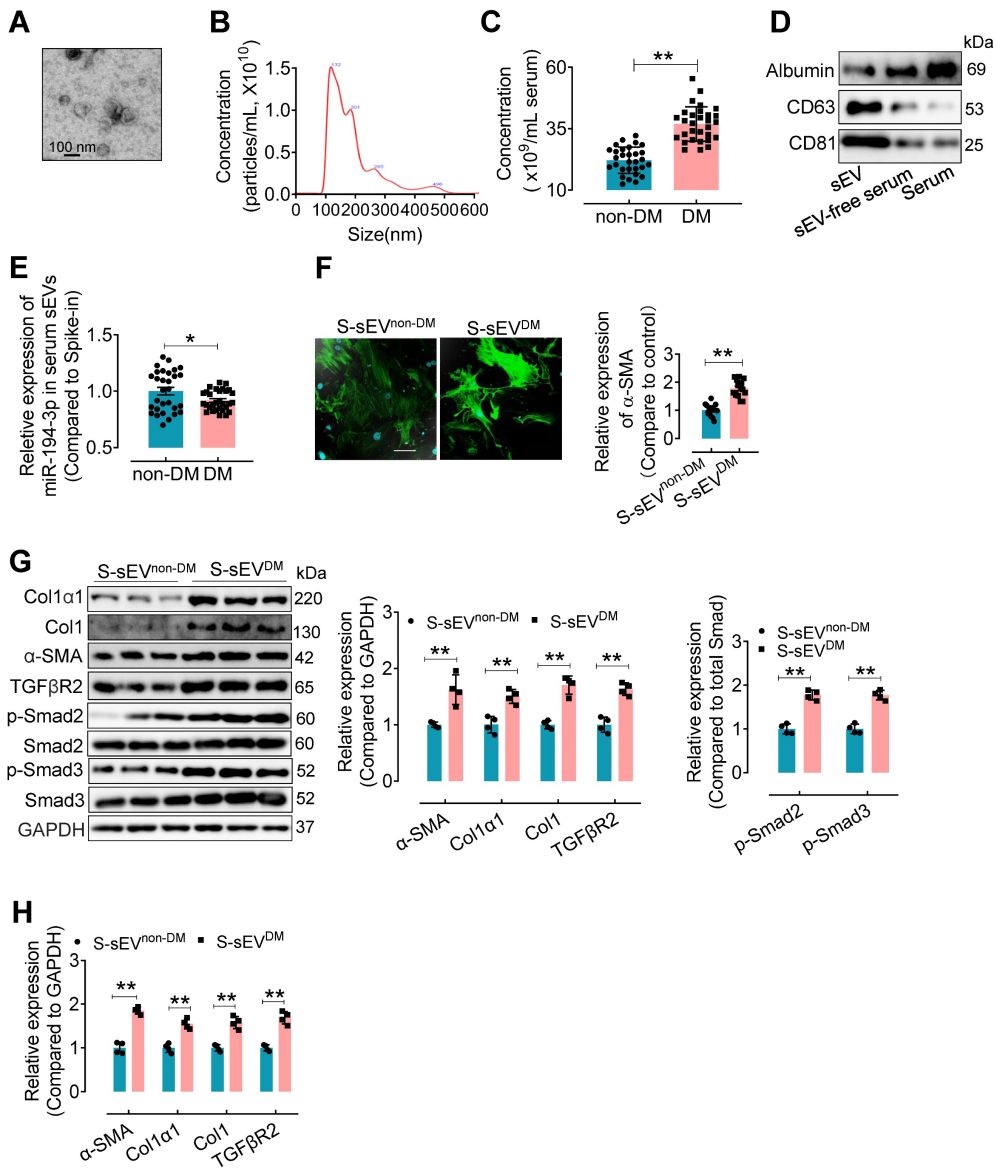
** Serum sEVs^DM^ contain lower miR-194-3p and induce cardiac fibroblast-to-myofibroblast conversion.** (**A**) The morphology of serum sEVs was characterized by TEM. Scale bars: 100 nm. (**B**) The size distribution of serum sEV was determined using Nanosight tracking analysis. (**C**) Nanosight tracking analysis demonstrated the concentration of serum sEVs from diabetic patients and non-diabetic patients. (n = 30). (**D**) Western blotting results showed the levels of albumin, CD63, and CD81 in the fractions of serum, sEV-free serum, and serum sEV. (**E**) miR-194-3p levels in serum sEVs derived from individuals with diabetes (S-sEV^DM^) and those without diabetes (S-sEV^non-DM^) (cel-miR-39 miRNA as a spike-in control, n = 30). (**F**) Immunostaining for α-SMA (green) showed a stronger signal in fibroblasts treated with S-sEV^DM^ compared to those treated with S-sEV^non-DM^. Scale bars: 20 μm. (n = 15). (**G, H**) The protein (H) and mRNA (I) expression levels of Col1α1, Col1, α-SMA, and molecules related to the TGFβ signaling pathway were assessed through qPCR and Western blotting in fibroblasts treated with S-sEV^non-DM^ or S-sEV^non-DM^. (n = 4). All values are presented as mean ± SEM. P values were calculated by unpaired two-tailed Student's t-test. *p < 0.05, **p < 0.01.

To confirm the clinical relevance, miR-194-3p levels in serum sEVs were evaluated in type 2 diabetes (T2DM) patients and non-T2DM subjects. Serum sEVs were isolated using ultracentrifugation from T2DM patients and non-T2DM individuals. The isolated serum sEVs exhibited a round and intact morphology with an average diameter of approximately 100 nm (Figure 8A and B). Compared to the non-T2DM group, the T2DM patients showed a significant increase in the number of serum sEVs (Figure 8C). Western blotting results revealed higher levels of sEV-associated proteins CD63 and CD81, but lower levels of albumin protein, in the serum sEVs fraction (Figure 8D). Subsequently, a qPCR assay showed that the levels of miR-194-3p were significantly decreased in serum sEVs from T2DM patients (S-sEVs^DM^) compared to non-DM individuals (S-sEVs^non-DM^) (Figure 8E). *In vitro* immunostaining experiments demonstrated that treatment with S-sEVs^DM^ led to an increased presence of α-SMA-positive myofibroblasts (green), compared to the treatment with S-sEVs^non-DM^ (Figure 8F). Importantly, the protein and mRNA expression levels of Col1α1, Col1, α-SMA, and TGFβR2 in fibroblasts were correspondingly upregulated by serum sEVs^DM^ treatment compared to the counterpart group (Figure 8G and H).

## Discussion

In our study, we made three important discoveries. First, our findings indicated that Myo-sEVs^Nor^ protected against diabetic cardiac fibrosis, whereas Myo-sEVs^HG/HL^ exacerbated it. Second, we observed a reduction in miR-194-3p levels in Myo-sEVs under diabetic conditions. Finally, we found that the introduction of a miR-194-3p (mimic or agomiR) successfully alleviated diabetic cardiac fibrosis and inhibited cardiac fibroblast-to-myofibroblast conversion by targeting the protein TGFβR2.

Numerous factors, such as oxidative stress, inflammatory mediation, cytokines, renin-angiotensin system, dysregulated glucose and lipid metabolism, end-glycation products and mitochondrial dysfunction 6, miRNA 22, and epigenetics 23, have been implicated in cardiac fibroblast-to-myofibroblast conversion. Recent research also highlighted the crucial role of the microenvironment in the process of cardiac fibrosis 9, 24. Cardiomyocytes and cardiac fibroblasts, the two major cell types in the heart, have extensive interconnectivity, allowing for effective communication through paracrine mediators 13. Among these mediators, cardiomyocyte extracellular vesicles (Myo-sEVs) have emerged as significant players in facilitating intercellular interactions. Unlike traditional models of cell communication that rely on ligand-receptor cross-talk, sEVs facilitate the function of recipient cells by delivering a diverse array of cargo. This comprehensive integration of various signaling pathways fosters intercellular cross-talk more intricately and efficiently 7. Recent studies have indicated that Myo-sEVs from ischemic cardiomyocytes promote the development of cardiac fibrosis under ischemic or heart failure conditions, whereas the Myo-sEVs from normal cardiomyocytes have a beneficial impact 14, 25. However, the exact influence of Myo-sEVs on diabetic cardiac fibrosis is still uncertain. Our present investigation offers conclusive and compelling evidence that Myo-sEVs from normal cardiomyocytes alleviate diabetic cardiac fibrosis, ameliorate diabetic cardiac dysfunction, and mitigate fibroblast-to-myofibroblast conversion induced by HG/HL conditions. Conversely, the uptake of Myo-sEVs^HG/HL^ or Myo-sEVs*^db/db^* by fibroblasts exacerbates diabetic cardiac fibrosis *in vivo* and increases α-SMA protein expression and fibroblast-to-myofibroblast conversion *in vitro*. Consistently, *in vivo* administration of Myo-sEVs^HG/HL^ or Myo-sEVs*^db/db^* enhances the expression of α-SMA, collagen Iα1, and type I collagen, and aggravates diastolic dysfunction in the hearts of diabetic mice. These results demonstrate, for the first time, the protective effect of Myo-sEVs derived from normal cardiomyocytes and the detrimental effect of Myo-sEVs^HG/HL^ or Myo-sEVs*^db/db^* on diabetic cardiac fibrosis. This will improve our understanding of the mechanisms underlying diabetic cardiac fibrosis and diabetic cardiomyopathy and provide insights into potential therapeutic strategies for diabetic cardiac fibrosis.

Recent studies have provided evidence showing the involvement of altered levels of miRNA within Myo-sEVs in the progression of cardiac fibrosis, particularly in the context of myocardial ischemia or heart failure. In mouse models of heart failure, Myo-sEVs enriched with miR-217 have been shown to contribute to fibroblast proliferation and the onset of cardiac hypertrophy 25. Notably, in addition to the increased levels of detrimental Myo-sEV miRNAs that facilitate cardiac fibrosis, there is also a concurrent reduction in certain intrinsic Myo-sEV miRNAs that offer protective effects against this condition. For instance, Myo-sEVs obtained from heart failure patients with reduced levels of miR-21-5p have been found to exacerbate cardiac dysfunction and contribute to left ventricular remodeling 14. In our study, we have identified miR-194-3p within Myo-sEVs as the critical endogenous molecule that protects against diabetic cardiac fibrosis, whereas its absence in diabetic Myo-sEVs promotes the development of this condition.

Non-coding RNAs, in particular the abundance of many miRNA passenger strands (“star” miRNAs), which are typically degraded intracellularly, are highly concentrated in sEVs 26. Notably, this study found that the "star" miRNA miR-194-3p was even higher in Myo-sEVs compared to the cellular source. Previous research has suggested that miR-194-3p contains a protein binding sequence (GGGCUG) for hnRNPQ, making it more likely to be sorted and enriched in extracellular vesicles 27. However, under conditions such as HG/HL treatment or diabetic conditions, the levels of miR-194-3p were significantly reduced in both Myo-sEVs and serum sEVs. More interestingly, premature pri-miR-194-3p showed minimal endogenous expression in primary cardiac fibroblasts compared to cardiomyocytes. Treatment with HG/HL significantly decreased miR-194-3p in cardiomyocytes and their corresponding Myo-sEVs, but did not affect miR-194-3p expression in primary cardiac fibroblasts. In contrast, treatment with Myo-sEVs^HG/HL^ markedly reduced miR-194-3p content in cardiac fibroblasts compared to Myo-sEVs^Nor^ treatment. These findings strongly support the notion that Myo-sEVs can transfer miR-194-3p from cardiomyocytes to cardiac fibroblasts. miR-194-3p has previously been reported to be implicated in cancer biology 28, and fibrotic lesions such as skin scarring 29 and liver fibrosis 30. Its role in cardiovascular physiology and pathophysiology is not well understood. This study is the first to report the critical role of miR-194-3p in diabetic cardiac fibrosis, demonstrating that the administration of miR-194-3p mimics attenuated Myo-sEVs^HG/HL^-induced fibroblast-to-myofibroblast conversion *in vitro*, and agomiR-194-3p improved diabetic cardiac fibrosis and cardiac function *in vivo*. These findings suggest that Myo-sEVs play a crucial role in mediating the protective effects of miR-194-3p from cardiomyocytes to cardiac fibroblasts through paracrine signaling pathways and highlight the therapeutic potential of targeting miR-194-3p as a therapeutic strategy for cardiac fibrosis, particularly in the context of diabetes.

Thirdly, our research has uncovered a critical role for miR-194-3p in protecting against diabetic cardiac fibrosis through the suppression of TGFβR2. Using extensive bioinformatic analysis and multiple *in vivo* and *in vitro* experimental validations, we have identified the TGFβ pathway as the primary pathway regulated by miR-194-3p, with TGFβR2 being a direct target gene of miR-194-3p. TGFβ is well-known for its ability to induce cardiac fibroblast-to-myofibroblast conversion, leading to the excess expression of α-SMA and the mass production of extracellular matrix, ultimately resulting in cardiac fibrosis. Recent research has shown that TGFβ upregulates miR-194-3p expression in embryonic epicardial cells, which mediates epithelial-mesenchymal transition by directly targeting and modulating p120-catenin expression 31. These results and our findings suggest that a negative feedback loop exists between miR-194-3p and the TGFβ pathway to fine-tune cellular TGFβ pathway activity and downstream phenotypes. Previous studies targeting TGFβ1 or TGFβR1 in cardiomyocytes have shown only modest improvements in cardiac fibrosis, with minimal reduction in ventricular dysfunction or myocardial hypertrophy. In contrast, knockdown TGFβR2 in both myocardial fibroblasts and cardiomyocytes has been shown to effectively inhibit both classical and non-classical TGFβ pathways, providing significant protection against afterload-induced cardiac fibrosis, myocardial hypertrophy, and ventricular dysfunction 32-34. Our study demonstrated that miR-194-3p mimic effectively suppressed the translational and transcriptional expression of TGFβR2. Conversely, Myo-sEVs^HG/HL^, serum sEVs from diabetic patients, and miR-194-3p inhibitors all promoted TGFβR2 expression. Furthermore, TGFβR2 inhibitors, such as SB431542, and TGFβR2 siRNA effectively decreased the expression of phosphorylated Smad2/3 induced by Myo-sEVs^HG/HL^ or miR-194-3p inhibitors. Under diabetic conditions, the excessive binding of TGFβ directly leads to a significant stimulation of TGFβR2, which is supported by robust clinical and experimental findings 35, 36. More importantly, animal studies have shown that competitive inhibition of TGFβR2 activity using soluble TGFβR2 can ameliorate diabetic nephropathy 37, implying that TGFβR2 is an important target for diabetic complications. Our study here provides a novel mechanistic explanation that diabetic Myo-sEVs elicit TGFβR2 signaling by delivering decreased levels of miR-194-3p to fibroblasts.

We have successfully established a link between cardiomyocytes and cardiac fibroblasts using Myo-sEVs in diabetes, elucidated the protective role of Myo-sEV^Nor^ in diabetic cardiac fibrosis, and the underlying mechanism of diabetic Myo-sEVs-induced cardiac fibroblast-to-myofibroblast conversion. However, there are still some critical issues that require further research. This study was conducted in male mice, and further confirmation is needed as to whether similar changes and effects occur in female mice. Although our focus was on the miRNA in Myo-sEVs, it is important to explore other unaddressed components such as proteins and lipids in Myo-sEVs, or other cell types and phenotypes that could be affected by Myo-sEVs, as they may also contribute to diabetic cardiomyopathy. Additionally, it is possible that miR-194-3p derived from non-Myo-sEVs may play a role in our system, albeit to a lesser extent, and this possibility requires further investigation. This novel form of cell-to-cell communication may also contribute to cardiac fibrosis in other pathogeneses, such as myocardial infarction and ischemic heart injury, which warrants additional investigation. Myo-sEVs could contribute to cardiac hypotrophy, as alterations in heart weight were observed following treatment with Myo-sEVs from various sources, and further studies are needed to explore this relationship and understand the mechanism. Finally, although we have validated that serum sEVs from diabetes patients contain lower levels of miR-194-3p compared to non-diabetic patients, it is crucial to conduct further in-depth clinical studies to determine whether these signatures can be used to predict diabetic cardiac fibrosis.

In summary, this study sheds light on the previously unexplored role of Myo-sEVs in regulating diabetic cardiac fibrosis, demonstrates that Myo-sEV miR-194-3p is the endogenous protective molecule of diabetic cardiac fibrosis, and its reduction in diabetic Myo-sEV is significantly associated with the development of cardiac fibrosis under diabetic conditions, by triggering the expression of TGFβR2 in fibroblasts. The study suggests that promoting miR-194-3p biogenesis or administering a miR-194-3p agonist may be a promising therapeutic approach against diabetes-related cardiac injury.

## Supplementary Material

Supplementary materials and methods, figures and tables.

## Abbreviations

CM: cardiomyocyte
FB: fibroblast
HFD: high-fat diet
HG/HL: high glucose/high fat
HW/TL: heart weight to tibia length ratio
LDH: lactate dehydrogenase
NC: negative control
ND: normal diet
NG/NL: normal glucose/normal fat
sEV: small extracellular vesicles
sEV^Con^: sEVs from primary cardiomyocytes of control mice
sEV*^db/db^*: sEVs from primary cardiomyocytes of *db/db* mice
sEV^Nor^: sEVs released from normal primary cardiomyocytes
sEV^HG/HL^: sEVs derived from HG/HL-treated cardiomyocytes
T2DM: type 2 diabetes mellitus
TGFβ1: transforming growth factor β1
TGFβR2: transforming growth factor β receptor 2
