# Supplementary material for: Cardiomyocyte-derived small extracellular vesicle: a new mechanism driving diabetic cardiac fibrosis and cardiomyopathy
Source: Theranostics. 2024 Sep 9;14(15):5926–44. doi: 10.7150/thno.99507 (PMC11426245; doi:10.7150/thno.99507)
Supplement: Supplementary file 1 — Supplementary materials and methods, figures and tables. [file thnov14p5926s1.pdf]

Cardiomyocyte-derived small extracellular vesicle: a new mechanism driving diabetic cardiac fibrosis and cardiomyopathy

Yu Li[1], Yunhui Du[2], Yang Liu[2], Xiuhuan Chen[1], Xinxin Li[2], Yanru Duan[3], Yanwen Qin[2], Huirong Liu[3], Xinliang Ma[4], Shaoping Nie[1]*, Huina Zhang[2]*

[1]Department of Cardiology, Beijing An Zhen Hospital, Capital Medical University, Beijing, 100029, China;
[2]Beijing An Zhen Hospital, Capital Medical University, The Key Laboratory of Remodeling Cardiovascular Diseases, Ministry of Education; Collaborative Innovation Center for Cardiovascular Disorders, Beijing Institute of Heart Lung and Blood Vessel Disease, Beijing, 100029, China;
[3]Department of Physiology & Pathophysiology, School of Basic Medical Sciences, Capital Medical University, Beijing, 100029, China;
[4]Department of Emergency Medicine, Thomas Jefferson University, Philadelphia, PA, PA19107, USA;

Short title: Extracellular vesicle in diabetic cardiac fibrosis

* Correspondence to:
Shaoping Nie, Center for Coronary Artery Disease, Division of Cardiology, Beijing Anzhen Hospital, Capital Medical University, Beijing, 100029, China. Tel.: +86-10-64456509, E-mail: spnie@ccmu.edu.cn
or
Huina Zhang, [2]Beijing An Zhen Hospital, Capital Medical University, The Key Laboratory of Remodeling Cardiovascular Diseases, Ministry of Education; Collaborative Innovation Center for Cardiovascular Disorders, Beijing Institute of Heart Lung and Blood Vessel Disease, Beijing, 100029, China. Tel, 0086-10-64456509, E-mail: whinnerzhn@mail.ccmu.edu.cn

Supplementary Materials and Methods

Clinical samples

The study design and protocol were approved by the Medicine Ethics Committee of Beijing Anzhen Hospital (2022036) and strictly followed the guidelines of the Declaration of Helsinki. 30 patients with type 2 diabetes mellitus (T2DM) and 30 healthy controls (non-diabetes) were recruited in the Department of Endocrinology and Health Management Center of Beijing AnZhen Hospital from June to November 2022 (Supplementary material online, *Table S1*). Written informed consent was obtained from all participants. All participants belonged to the Han nationality. Diagnosis of T2DM in patients was based on the American Diabetes Association criteria [1], which includes a previous diagnosis of T2DM, fasting blood glucose ≥ 7mM, or 2-hour plasma glucose ≥ 11.1 mM during 75 g oral glucose tolerance test, or random glucose concentrations ≥ 11.1 mM, or HbA1c ≥ 6.5%, or patients being on glucose-lowering treatment for diabetes. Subjects with fasting blood glucose < 6 mM were enrolled in the non-diabetes group. Exclusion criteria comprised type I diabetes mellitus, serious cardiovascular disease (CVD, cardiac function level at III or IV by New York Heart Association standards), severe hepatic and renal dysfunction, active liver diseases, malignancy, inflammatory process in the past two months, pregnancy, or any factor affecting body weight such as hyperthyroidism or corticosteroids. Fasting serum samples were collected and stored at -80 ℃. Clinical test data were retrieved from hospital databases and analyzed.

Animal studies

All animal experiments were approved by the Capital Medical University Animal Experimentation Ethics Committee, and in compliance with the National Institutes of Health Guidelines on the Use of Laboratory Animals. High-fat diet (HFD) with low-dose streptozotocin (STZ) injection-induced and genetic (*db/db*) type 2 diabetic models were utilized in this study. 8-week old male C57BL/6 mice were purchased from SBF Biotech Co., Ltd. (Beijing, China) and fed with a high-fat diet (HFD, 60% fat, SYSE Bio-Tec. Co., Changzhou, China) for 8 weeks to induce obesity. Then, a diabetic mice model was constructed with small doses of STZ treatment. Briefly, STZ (dissolved in 50 mM sodium citrate buffer (pH 4.5) to a final concentration of 40 mg/mL) was administered intraperitoneally to the experimental group at a dose of 40 mg/kg. An equal volume of citrate buffer (pH 4.5) was injected intraperitoneally into the control animals. 5 days after administration of STZ, blood glucose levels were measured in tail vein blood samples to evaluate the diabetic model. STZ-treated mice always maintained a hyperglycemic state (> 15 mM (270 mg/dL)) for several weeks. A diabetic cardiac fibrosis mouse model was obtained by continuing a high-fat diet (HFD) for 22 weeks throughout the modeling process [2]. Approximately 6-8-week-old male *db/db* mice were purchased from Junke Biology Co., Ltd. (Nanjing, China), and the same aged C57BL/6 male mice were treated as control. The animals were maintained in a temperature-controlled (22 ± 2 °C) room with a 12 h light/dark cycle and free access to food and water. 20-22-week-old *db/db* mice and their controls were admitted to the experiment.

Measurement of blood pressure by tail-cuff plethysmography

The Softron BP2010A system (Beijing, China) for noninvasive blood pressure monitoring in mice was utilized to assess blood pressure by tracking changes in tail volume. The blood pressure measurements were conducted in a designated, quiet area maintained at 22 ± 2°C. Mice were allowed to acclimatize for an hour before the experiments commenced then gently encouraged to enter the restraint tubes, and the tube end holders were adjusted to minimize excessive movement. The occlusion cuff was positioned at the base of the tail, and the VPR sensor cuff was placed adjacent to it. The mice were warmed for 5 min on heating pads (prewarmed to a temperature range of 33 °C to 35 °C) before and throughout the blood pressure recordings. To assess blood pressure, the occlusion cuff was inflated to 250 mmHg and then gradually deflated over 20 s. As the blood returned to the tail during the deflation of the occlusion cuff, the VPR sensor cuff detected changes in tail volume. Each recording session comprised 15 to 25 inflation and deflation cycles per set. The initial five cycles served as "acclimation" cycles and were excluded from the analysis, while the subsequent cycles were utilized. Mice underwent a habituation period of at least five consecutive days before baseline blood pressure measurements.

Cardiomyocyte isolation and culture

C57BL/6 male mouse primary cardiomyocytes were isolated following a previously established protocol[3]. Briefly, adult male C57BL/6 mice (8 weeks) were anesthetized with 2% isoflurane followed by cervical dislocation. The mouse hearts were removed and retrogradely perfused through an aortic cannula at 37°C using a Langendorf apparatus (WPI, United States) with a constant flow rate of 4.5 mL/min perfusion buffer (116 mM NaCl, 5.4 mM KCl, 6.7 mM $MgCl_2$, 12 mM glucose, 2 mM glutamine, 3.5 mM $NaHCO_3$, 1.5 mM KH_2PO_4,1.0 mM NaH_2PO_4, 21 mM HEPES) for 5 min. Subsequently, the hearts underwent a second perfusion with a digestion solution supplemented with 0.6 mg/mL collagenase II (Invitrogen, Paisley, Renfrewshire, UK) and 15 mM $CaCl_2$ at 37°C for 10 min. Afterward, the ventricular tissue was quickly minced into pieces smaller than 1 mm^3 using fine forceps. The cell suspension was then transferred to a 15 mL conical tube, allowed to settle by gravity for 15 min, and carefully separated with a transfer pipette to cell culture dishes until only 50-100 μL of solution remained above the tissue fragments. The cell culture dish was promptly moved to a 37 °C incubator with 5% CO_2 and 95% humidity. Cardiomyocytes exposed to 5 mM glucose were categorized as the Control group, while those treated with 25 mM glucose plus 250 μM palmitate for 24 h were designated as the high glucose/high lipid (HG/HL) group.

Cardiac fibroblast isolation and culture

C57BL/6 male mouse primary cardiac fibroblast were isolated as described previously[4]. Briefly, adult male C57BL/6 mice (8 weeks) were anesthetized with 2% isoflurane followed by cervical dislocation. The hearts were isolated under sterile conditions and placed in Hank's balanced salt solution (HBSS, Corning, USA), supplemented with 100 U/mL penicillin and 100 μg/mL streptomycin (Corning, USA) and 0.01 mM HEPES (Sigma, USA). After rinsing again with fresh HBSS, the hearts were transferred into a sterile culture dish containing DMEM (Gibco, USA) supplemented with 0.25 mg/mL collagenase type II and 0.01 mM HEPES. The hearts were then minced into 1 mm^3 pieces and incubated on an orbital shaker at 37 °C for 35 min with the collagenase solution. Then, 25 mL ice-cold HBSS was added to inhibit collagenase activity. The dispersed cells

were separated from undigested tissues by passing through a 40-μm-diameter cell strainer and collected by centrifugation at 1,000 rpm for 5 min at 4 °C. After a single wash in HBSS by centrifugation, cells were resuspended with 10 mL DMEM supplemented with 10% fetal bovine serum (FBS, Gibco), 100 U/mL penicillin, and 100 μg/mL streptomycin, and seeded into sterile culture dishes. The cells were then cultured in a humidified atmosphere with 5% CO_2 at 37°C for 2 h. Afterward, the unaffixed cells were removed by changing to a fresh medium and the affixed cells were purified fibroblasts.

Glucose tolerance and insulin tolerance tests

To assess glucose tolerance, mice were intraperitoneally injected with D-glucose (1.5 g/kg) after an overnight fast of 16 h with free access to water. Blood glucose levels were measured from the tail vein using a glucometer and strips (ACCU-CHEK, Roche) at 0, 15, 30, 60, and 120 min after glucose injection as reported [5]. The insulin tolerance test was performed by measuring blood glucose level after 6 h of fasting, followed by intraperitoneal injection of 0.5 U/kg insulin (Novolin R) at 0, 15, 30, 60, and 120 min.

Cell viability assay

Cell viability was assessed using an MTT (3-[4,5-dimethylthiazol-2-yl]-2,5-diphenyltetrazolium bromide, Sigma M2128) assay. Briefly, cells were seeded into a 96-well plate and MTT solution was added to achieve a final concentration of 0.5 mg/mL. After a 4 h incubation, the media were gently removed without disrupting the cells, and 100 μL of DMSO was added to each well. The absorbance values were measured at 570 nm using a multi-well spectrophotometer (SpectraMax M5, Molecular Devices). Each experiment was performed in sextuplicate, and the relative cell viability (%) was expressed as a percentage relative to the control group cells.

Lactate dehydrogenase (LDH) releasing analysis

LDH levels (an indicator of cell injury) were measured using the LDH Cytotoxicity Detection kit (Takara, MK401) following manufacturer protocol. 100 μL of the supernatant was mixed with an equal volume of pre-prepared solution (catalyst/dye buffer ratio=1:45) for 30 min at room temperature. The absorbance values at 490 nm were read using a multi-well spectrophotometer SpectraMax M5 (Molecular Devices).

Analyses of cardiomyocyte and fibroblast purity

Cardiomyocyte and fibroblast purity were assessed using flow cytometry. Data acquisition was done by BD LSRFortessa Flow Cytometer (BD Biosciences) and data were analyzed with FlowJo Software (BD Biosciences). Briefly, for cardiomyocyte purity analysis, flow cytometric evaluations were performed according to the intracellular staining of cTnT (Cardiac troponin T, Fluor488, ABclonal, China), and nucleated cells were identified by propidium iodide (PI, HY-D0815, Medchemexpress LLC, China)[6]. For fibroblast purity analysis, flow cytometric evaluations were performed by $CD45^-$ (Fluor488, ABclonal, China), $CD31^-$ (Fluor594, ABclonal, China), and mEF-$SK4^+$ (APC, ABclonal, China)[7].

Echocardiography

Transthoracic echocardiography in mice was performed using Vevo 2100 (VisualSonics). Briefly, mice were anesthetized initially with 2 % isoflurane and securely positioned on a platform. Recordings were obtained from the Parasternal Long Axis View (PLAX) and Parasternal Short Axis View (PSAX) of the left ventricle (LV) in

both B and M modes. Mitral valve flow and mitral valve annulus velocities were evaluated using pulsed-wave and tissue Doppler imaging, respectively, from the apical four-chamber view. Echo data was analyzed using the Vevo LAB software (VisualSonics). All measurements were averaged from three cardiac cycles per animal at comparable heart rates. To assess wall global longitudinal strain (GLS) and regional peak longitudinal strains of the heart, the VevoStrain software (VisualSonics) was used. The representative Doppler images were at the different scales in the panels as indicated.

Myo-sEV purification and identification

Myo-sEVs were isolated from the culture media of cardiomyocytes by ultracentrifugation as previously described method[8]. Briefly, the culture medium of cardiomyocytes exposed to normal or high glucose/high lipid (HG/HL) conditions was collected and subjected to a series of centrifugation steps: 300 × g for 15 min, and 12,000 × g for 30 min, aimed at removing cardiomyocytes and cellular debris. Subsequently, the supernatant was filtered through a 0.2 μm microporous membrane (Millipore, MA, USA) and further subjected to ultracentrifugation at 110,000 × g for 120 min (Beckman Coulter, CA, USA). The sEV pellets were washed with PBS to eliminate contaminating proteins, followed by a second round of ultracentrifugation at 110,000 × g for 90 min All centrifugation steps were performed at 4 °C. The characterization of sEVs was confirmed by measuring the expression of sEV-specific markers, such as CD63, CD81, TSG101, Alix, calnexin, and cardiomyocyte-specific markers α-sarcomeric actin *via* Western blotting analysis. The particle size and number were detected by NanoSight analysis (NS300, Malvern Instruments), and the sEV morphology was visualized using a transmission electron microscope (TEM, H-600, Hitachi, Japan) with negative staining [8].

Serum sEV purification

Human or mouse sera were first diluted with PBS and then subjected to a series of centrifugation steps: low-speed centrifugation at 300 × g for 15 min and subsequently at 12,000 × g for 30 min, all performed at 4 °C. The resulting supernatants were filtered through a 0.22 μm filter (Merck, Darmstadt, Germany). The supernatant was then processed by ultracentrifugation at 100,000 × g for 90 min at 4 °C using a T-70i fixed-angle rotor (Beckman Coulter, Brea, CA) to pellet the sEVs. The pellet was subsequently washed with PBS to remove any remaining contaminating proteins and subjected to another round of ultracentrifugation under the same conditions. Finally, the supernatant was discarded, and the sEV pellet was collected.

Myo-sEV treatment, labeling, and absorption detection

We employed two methods to assess the impact of *in vivo* treatment with Myo-sEVs in mice. The first approach involved utilizing sEVs ($1\sim5\times10^{10}$) secreted by an equivalent number of cardiomyocytes (10^6, with different treatments). The second approach involved utilizing an equivalent quantity of sEVs (1.2×10^{10}). Additionally, we treated the cells with sEVs ($1\sim5\times10^9$) secreted by an equivalent number of cardiomyocytes (10^5, with different treatments) or with different doses of sEVs (0, 1×10^9, $2\times10^{9,}$ and 4×10^9/well) to evaluate their effects *in vitro*. To monitor Myo-sEVs trafficking in vitro, Myo-sEVs were labeled with PKH67 fluorescent dye using the PKH67 Fluorescent Cell Linker Kit (Sigma-Aldrich) following the manufacturer's protocol. The PKH76-labeled Myo-sEVs were incubated with fibroblasts for various time intervals (0, 1, 4, 12, 24, and 48 h/well) and various

dose intervals (1×10^9, $2\times10^{9,}$ and 4×10^9/well). Afterward, the cells separately were analyzed through western blotting and immunofluorescence. The cells were fixed with 4 % paraformaldehyde for 10 min at room temperature, washed twice with PBS, and stained with 1 μg/mL DAPI (4’,6-diamidino-2’-phenylindole, dihydrochloride, sigma, D9542) for 5 min at room temperature. Then the fluorescence labeled-Myo-sEVs were utilized to treat fibroblasts or C57BL/6 mice for different periods, and the signal of Myo-sEVs was visualized under a confocal microscope (FV1000; Olympus).

Myo-sEVs intramyocardial and systemic delivery

PKH67 labeled Myo-sEVs were intramyocardially injected into three different sites of the left ventricle (LV) after the mice were anesthetized with 2% isoflurane as previously described[9]. Simply, a small skin cut (1.2 cm) was made over the left chest of the mice, and a purse suture was made. After dissection and retraction of the pectoral major and minor muscle, the fourth intercostal space was exposed. A small hole was made at the fourth intercostal space with a mosquito clamp to open the pleural membrane and pericardium. With the clamp slightly open, the heart was smoothly and gently “popped out” through the hole. Immediately PKH67 labeled Myo-sEVs (approximately $1\sim5\times10^{10}$, equivalent to about 50 μg Myo-sEVs diluted in 30 μL PBS) were intramyocardially injected into three different sites of the left ventricle (LV). Sham control animals were subjected to all surgical procedures, except for the injection of 30 μL PBS. All mice received an intraperitoneal injection of cyclosporine A (10 mg/kg per day, Signa-Aldrich) 2 days before transplantation, then daily until the end of the study. 48 h after the injection (keeping analgesia with ketoprofen (100 mg/kg)), the heart was isolated and sectioned for laser confocal microscopy. Myo-sEVs (approximately $1\sim5\times10^{10}$, equivalent to about 50 μg Myo-sEVs diluted in 50 μL PBS) were also administered to both non-diabetic and diabetic mice using a double tail-vein injection method, with a 7-day interval between injections.

Western blotting

Protein was extracted using a protein extraction kit (BC3710, Solarbio, Beijing, China), and protein concentration was quantified using a BCA assay (Thermo Fisher Scientific, USA) according to the manufacturer's instructions. Subsequently, 5-10 μg of protein was separated by 10% - 12% SDS-PAGE and transferred onto a nitrocellulose membrane (EMD Millipore, USA). After blocked with 5% nonfat milk, the membranes were incubated with primary antibodies overnight at 4˚C. Then, the membranes were incubated with horseradish peroxidase-conjugated secondary antibodies (1:5,000; K5007; Dako, USA) for 1 h at room temperature. The protein bands were visualized using the SuperSignal chemiluminescent detection module (34080; Pierce), and images were captured using a ChemiDoc™ Touch (Bio-Rad, USA). The primary antibody information can be found in the Supplementary material online, *Table S2*.

Transmission electron microscopy

The morphology of sEVs was examined using a transmission electron microscope (TEM, Hitachi H-7650) to observe their ultrastructure, following established procedures [8]. Briefly, the sEV pellets were fixed with 2% PFA and deposited onto EM grids. Subsequently, the grids were separately transferred into 1% glutaraldehyde

and methyl cellulose-UA for 10 minutes. After air drying, the grids were examined using the FEI Tecnai electron microscope at 80 kV.

miRNA library preparation, microarray chip assay, and data analysis

sEVs for miRNA microarray chip assay obtained from 50 mL culture medium of C57BL/6 mouse primary cardiomyocytes (from 5 mice) were collected and frozen at -80°C as one sample. sEV miRNAs were extracted by using the miRNA isolation Kit (AM1561, Thermo Fisher Scientific, USA) according to the manufacturer's protocol. MicroRNA array analysis was conducted by Oebiotech Co., Ltd. (Shanghai, China). Briefly, 100 ng total miRNA per sample served as an input material for the small RNA library preparation. The libraries were generated using NEBNext® Multiplex Small RNA Library Prep Set for Illumina® (NEB, E7300L). The library quality was assessed with the Agilent Bioanalyzer 2100 system using miRNA High Sensitivity Chips. Subsequently, the chip was sequenced on an Agilent Mouse miRNA 21.0 platform. Known miRNAs were identified by comparing them to the miRBase 21.0 database (http://www.mirbase.org/). The default criteria for significantly differential expression were set as p-value < 0.05 and $| \log 2$ (fold change) $| > 1$. Differential miRNAs were exhibited by volcano plot and hierarchical clustering heatmap. miRNA array data have been deposited in ArrayExpress under accession number E-MTAB-13736.

Transfection of miRNA mimics and inhibitors into fibroblast or delivery of miRNA agomiR by tail vein injection

The miR-194-3p mimic, miR-194-3p inhibitors, and their respective negative controls (NC) (Gene Pharma, China) were mixed with TransMessenger Transfection Reagent (Qiagen, 301525) and administered to fibroblasts for 4 h following the manufacturer's protocol. Subsequently, the culture media were replaced with complete culture media devoid of antibiotics. After 24 h of transfection, the cells were exposed to HG/HL plus Myo-sEVs for an additional 24 h. For the *in vivo* experiment, a mixture of 10 nM agomiR-194-3p (Ribibio, China, miR40017148-4-5) and its negative controls (NC) was prepared in 50 µL PBS buffer. This mixture was then administered *via* tail vein injection, with two injections spaced one week apart.

Reporter gene assay

The HEK293 cells treated with 10 pM miR-194-3p mimic or NC mimic were transfected with 0.4 µg or 0.2 µg of the respective reporter plasmids or the corresponding empty vector (pGL3-Promoter) as a control, along with 20 ng of pRL-SV40 as an internal control reporter. After 48 hours post-transfection, the activities of firefly and renilla luciferases were detected using the Dual-Glo^{TM} Luciferase Assay System (Promega, E2920) following the manufacturer's instructions. The absorbance values were recorded using a multi-well spectrophotometer SpectraMax M5 (Molecular Devices). All experiments were performed in triplicate and repeated at least three times.

Isolation of miRNAs and total RNAs, and detection of miRNAs, mRNA, and pri-miRNAs

The sEV-containing small RNAs were extracted using the Total Exosome RNA & Protein Isolation Kit (AM1561, Thermo Fisher Scientific, USA) or the exoRNeasy Serum/Plasma Starter Kit (Qiagen, 77023, USA) following the manufacturer's protocol. The expression of 15 mature miRNAs including mmu-miR-30c

(mmu483230_mir), mmu-miR-143-5p (mmu480936_mir), mmu-miR-181b-5p (245498_mat), mmu-miR-193-3p (002250), mmu-miR-194-5p (000493), mmu-miR194-3p (002379), mmu-miR-214-5p (002293), mmu-miR-448-5p (464921_mat), mmu-miR-539-5p (001286), mmu-miR-574-5p (002349), mmu-miR-466g (241015_mat), mmu-miR-704 (001639), mmu-miR-1198-5p (002780), U6 snRNA (001973), cel-miR-39 (000200) was detected by the TaqMan™ MicroRNA Assay (Thermos Fisher, 4427975) using the TaqMan MicroRNA Reverse Transcription Kit (Thermo Fisher, 4366596) and Taqman Fast Advanced Master Mix (Thermo Fisher,4444557). U6 snRNA served as a reference control in sEVs derived from cells and tissues. In the case of sEVs from serum, where U6 snRNA might not be encased within sEVs equally, cel-miR-39 miRNA (Qiagen, 219610) was used as a spike-in control. Total RNAs from mouse tissues and primary fibroblasts were isolated using the TRIzol reagent (Invitrogen, 15596018) according to the manufacturer's protocol. The 10 pri-miRNAs were detected using Pri-miRNA Assay (*Pri-miR-181-1, Pri-miR-181-2, Pri-miR-194*, *Pri-miR-539, Pri-miR-574*, *Pri-miR-143*; GenePharma, E22001, China), with 18s RNA serving as a reference control. Transcripts of genes, including pri-miRs and predicted target genes, were detected using RevertAid First Strand cDNA Synthesis Kit (ThermoFisher, K1622) and PowerUp™ SYBR® Green Master Mix (ThermoFisher, A25742) on a QuantStudio Real-Time PCR System (ABI,7500) in a 96-well format, respectively. The primer details were available in the online supplement (Supplementary material online, *Table S3*).

Histology and fluorescent immunostaining

Hematoxylin and eosin (H&E) staining was performed in the sections of mouse cardiac tissue. Mouse cardiac tissues were fixed in 4% paraformaldehyde, and embedded in paraffin wax, and 4 μm thick slices were cut. After deparaffinization at 60°C, the sections were incubated twice in xylene, and dehydrated in gradient ethanol solutions. The sections were stained for 10 minutes with Harris hematoxylin staining solution followed by incubation with 1% HCl in ethanol for 30 seconds. The slices were then washed with tap water for 15 minutes, stained with 1% eosin iron-red, and incubated with 90% ethanol. Then, the sections were washed with 95% ethanol for 1 minute, and then 3 times with xylene. Finally, the hematoxylin and eosin-stained sections were allowed to sit at room temperature for 20 minutes and then analyzed by light microscopy to determine the morphological changes.

Picrosirius red staining as the fibrosis staining was performed using the Picrosirius Red Stain Kit (Polysciences, Inc. Warrington, PA). Briefly, paraffin sections were de-waxed and hydrated. Nuclei were stained with Weigert's hematoxylin and washed with running tap water. Picrosrius stain was applied to the tissue slides for one hour, followed by washing in two changes of acidified water. All water was removed and the tissue samples were dehydrated using three changes of 100% ethanol. The tissue was then cleared in xylene and mounted in a resinous medium. Images were captured at 20 × magnification with a Nikon E800 Eclipse microscope (Nikon, Tokyo, Japan) with the same exposure in 5 random fields. Image J software (National Institutes of Health, Bethesda, MD) was used to measure collagen expression in a blinded fashion. Representative images have been included in the manuscript.

Cultured fibroblasts were fixed in 3.7% formaldehyde solution (Sigma) in PBS for 10 minutes and then permeabilized using 0.1% Triton-100 (Sigma). Subsequently, cells were incubated with anti-α-SMA antibody (Abcam, ab7817) for 3 hours at room temperature (RT) or overnight at 4°C. Nuclei were stained using DAPI (ZSGB-bio, China).

Statistical analyses

Statistical data were presented as mean ± SEM (standard error of the mean). GraphPad Prism version 8.0 and SAS (Statistical Analysis System) were used for all statistical analyses except for miRNA chip data. Unpaired two-tailed Student's t-test was used to compare two groups; one-way analysis of variance (ANOVA) followed by Tukey's multiple comparisons test was used to compare differences among > 2 groups. All of the statistical experiments were repeated at least three times independently. $P < 0.05$ was considered statistically significant. The representative image was selected from one of the repeated experiments that best matched the mean value.

Supplementary Figures

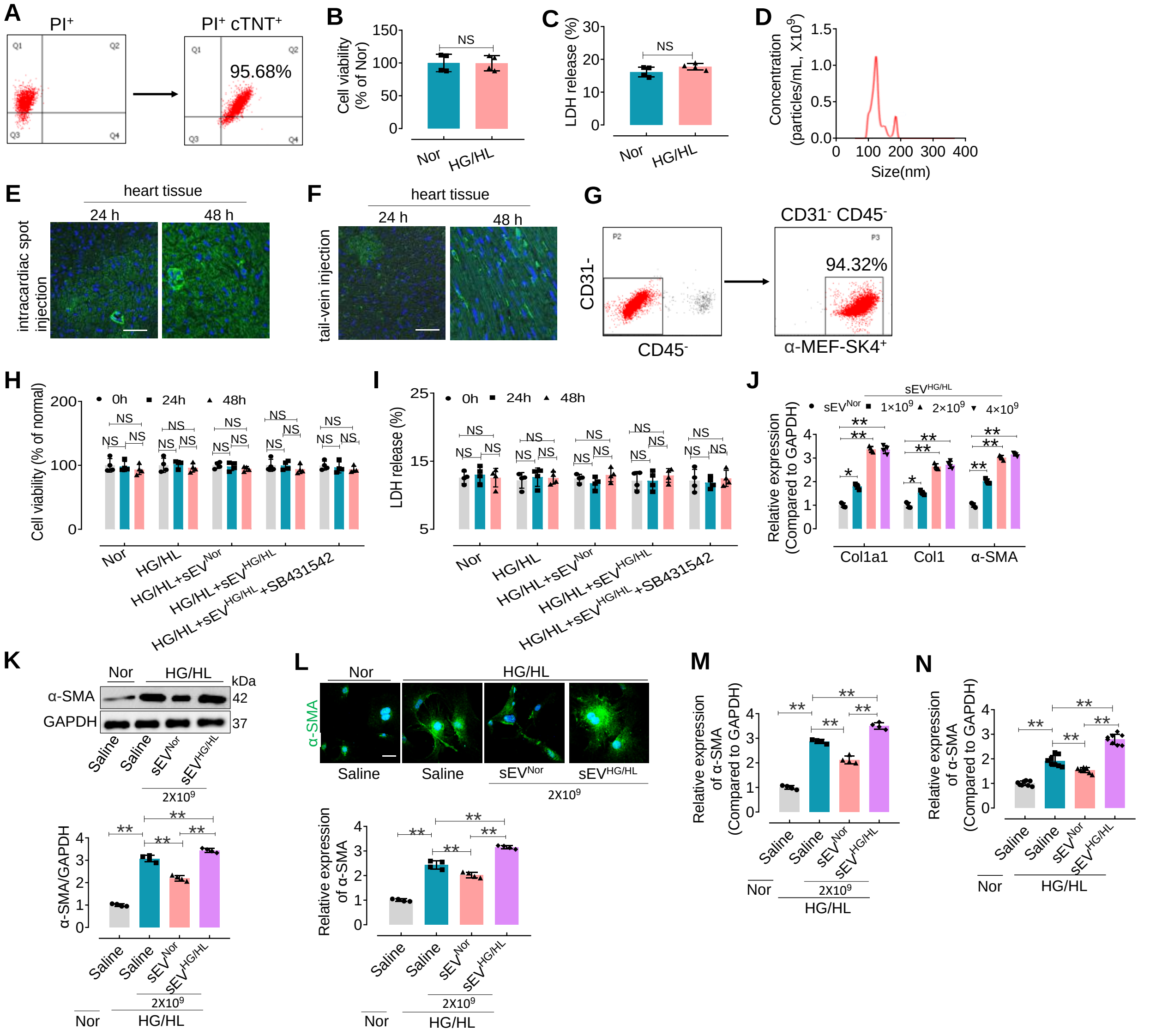

Supplementary Figure 1. The assessment of primary cardiomyocyte purity, viability, the uptake of Myo-sEVs by heart tissue *in vivo*, and the cell viability assay of fibroblasts after uptake of indicated Myo-sEVs. (A) Representative flow cytometry data evaluated the percentage of cardiomyocytes (PI^+ $cTNT^+$) among nucleated cells (propidium iodide, PI). The purity of cardiomyocytes was 95.68% ± 2.58 (n = 3)**. (B, C)** Primary cardiomyocyte viability and cell survival were evaluated by MTT assay (B) or LDH release assay (C) after in vitro treatment with normal glucose/normal lipid (Nor) or high glucose/high lipid (HG/HL, 25 mM glucose plus 250 μM palmitate) for 24 h (n = 4). **(D)** The Myo-sEVs particle size distribution was determined by Nanosight tracking analysis. **(E, F)** In vivo intramyocardial injection (E) or (F) tail vein injection of PKH67-labeled Myo-sEVs showed notable fluorescent signals, indicating successful uptake of the vesicles by mouse heart cells. **(G)** Flow cytometry analysis showed the purity of primary fibroblasts. Fibroblasts were defined as $CD45^-$ $CD31^-$ $MEF\text{-}SK4^+$. The purity of fibroblasts is 94.32% ± 3.31 (n = 3). **(H, I)** Fibroblast viability and survival were determined by MTT assay (H) and LDH release assay (I) after in vitro treatment with Myo-sEV^{Nor}, Myo-$sEV^{HG/HL}$ or SB431542. (n = 4). **(J)** qPCR analysis result showed the transcriptional expression of Collagen1α1, Collage1, and α-SMA in fibroblasts under different Myo-$sEV^{HG/HL}$ gradient dose (1×10^9, 2×10^9 and 4×10^9) treatment with Myo-sEV^{Nor} (1×10^9) as the control. (n = 4). **(K, L)** The protein expression of α-SMA was detected in fibroblasts under 24 h treatment with normal glucose/normal lipid (Nor) or HG/HL, followed by Myo-$sEVs^{Nor}$ (2×10^9) or Myo-$sEVs^{HG/HL}$ (2×10^9) for an additional 24 h by Western blotting (K) or immunostaining (green) (L). (n = 4) Scale bars: 20 μm. **(M)** The transcriptional levels of α-SMA were detected in fibroblasts under 24 h treatment with normal glucose/normal lipid or HG/HL, followed by Myo-$sEVs^{Nor}$ (2×10^9) or Myo-$sEVs^{HG/HL}$ (2×10^9) for an additional 24 h. (n = 4). **(N)** The transcriptional levels of α-SMA were detected in fibroblasts under 24 h treatment with normal glucose/normal lipid (Nor) or HG/HL, followed by Myo-$sEVs^{Nor}$ or Myo-$sEVs^{HG/HL}$ from an equal number of cardiomyocytes (10^5) for an additional 24 h**.** All values are presented as mean ± SEM. P values were calculated by unpaired two-tailed Student's t-test (B, C) or one-way ANOVA followed by Tukey's test (H-N). *$p < 0.05$, **$p < 0.01$.

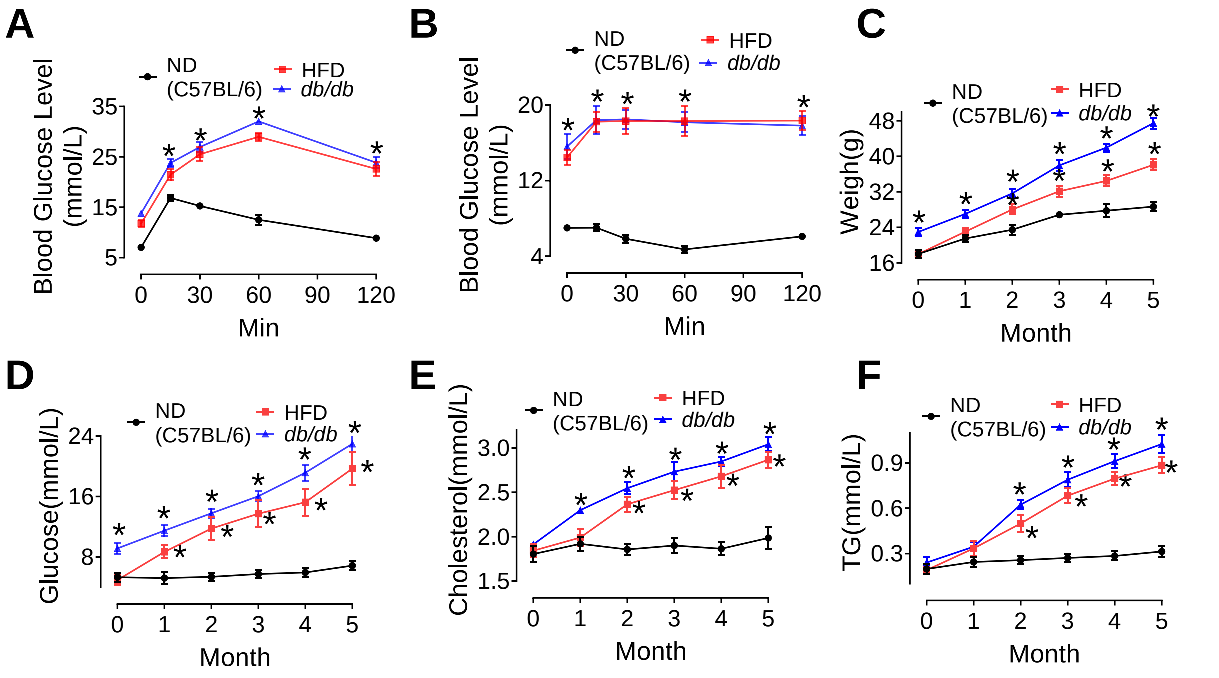

Supplementary Figure 2. Evaluation of diabetic mouse model. (A, B) GTT and ITT tests were performed in HFD-induced diabetic mice (A) or *db/db* mice (B) (n = 8, *p < 0.05). **(C-F)** Physiological and biochemical indexes including body weight (C), glucose (D), cholesterol (E), and triglyceride (TG) (F) during 5 months in mice with HFD-induced diabetic mice, *db/db* mice and their counterpart group. (n = 10, *p < 0.05). All results are presented as mean ± SEM. P values were calculated by one-way ANOVA, Tukey's multiple comparisons test.

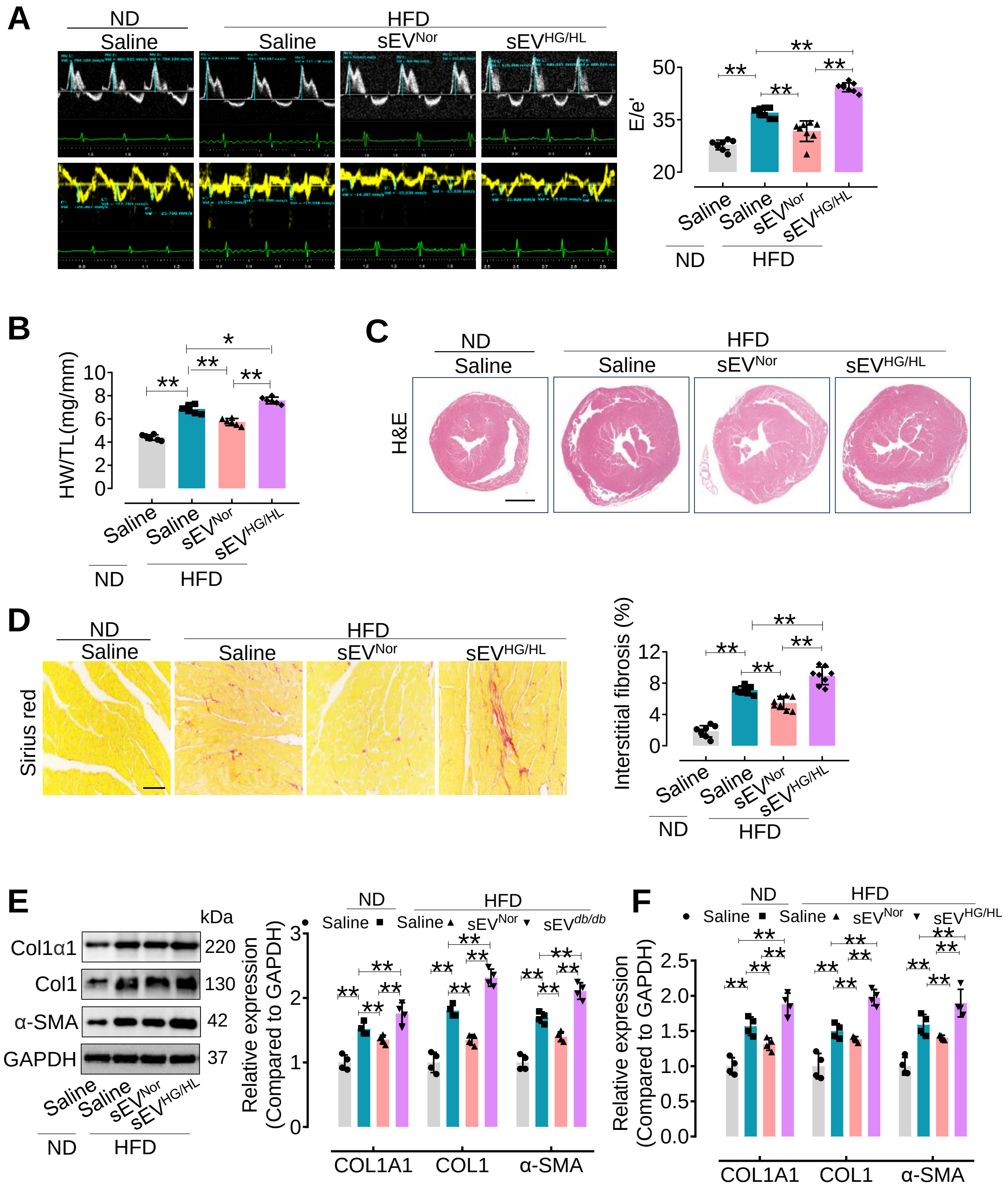

A
ND
Saline
HFD
Saline
sEVNor
sEVHG/HL
E/e'
50
35
20
**
Saline
Saline
sEVNor
sEVHG/HL
ND
HFD
B
HW/TL(mg/mm)
10
8
6
4
2
0
*
C
H&E
D
Sirius red
Interstitial fibrosis (%)
12
8
4
0
E
kDa
Col1α1
220
Col1
130
α-SMA
42
GAPDH
37
Relative expression
(Compared to GAPDH)
sEVdb/db
COL1A1
COL1
α-SMA
3
2
1
0
F
2.5
2.0
1.5
1.0
0.5
0.0

Supplementary Figure 3. The function of the same number of Myo-sEV^{Nor} or Myo-sEV $^{HG/HL}$ (1.2×10^{10}) on myocardial fibrosis in HFD plus STZ-induced diabetic mice. (A) E-waves (upper panel) were measured using pulsed wave (PW) Doppler from a 4-chamber view of the lateral mitral valve. Early diastolic (e') velocities were obtained from the Tissue Doppler signal of the mitral annulus (lower panel). The ratio of peak E to peak e' tended to increase significantly after treatment with Myo-sEVs$^{HG/HL}$ compared to Myo-sEVs^{Nor}. (n = 8). **(B)** The hearts and tibia length (mm) ratio (HW/TL) in HFD mice increased when treated with Myo-sEVs$^{HG/HL}$ compared to Myo-sEVs^{Nor}. (n = 8). **(C)** Hematoxylin and eosin staining of whole mouse heart histological sections were performed after *in vivo* treatment of ND or HFD mice using Myo-sEV^{Nor} and Myo-sEV$^{HG/HL}$. Scale bar: 1 mm. **(D)** Representative images of histological sections of mouse hearts and quantification of Picrosirius red-stained areas showing collagen deposition after the *in vivo* treatment of ND or HFD mice with Myo-sEV^{Nor} and Myo-sEV$^{HG/HL}$ (n = 8). Scale bars: 100 μm. (**E, F)** The protein (E) and mRNA (F) expression levels of Col1α1, Col1, and α-SMA were assessed through Western blotting and qPCR separately in the hearts of mice subjected to the indicated treatment. (n = 4). All values are presented as mean ± SEM. P values were calculated by one-way ANOVA, and Tukey's multiple comparisons test. $^*p < 0.05$, $^{**}p < 0.01$.

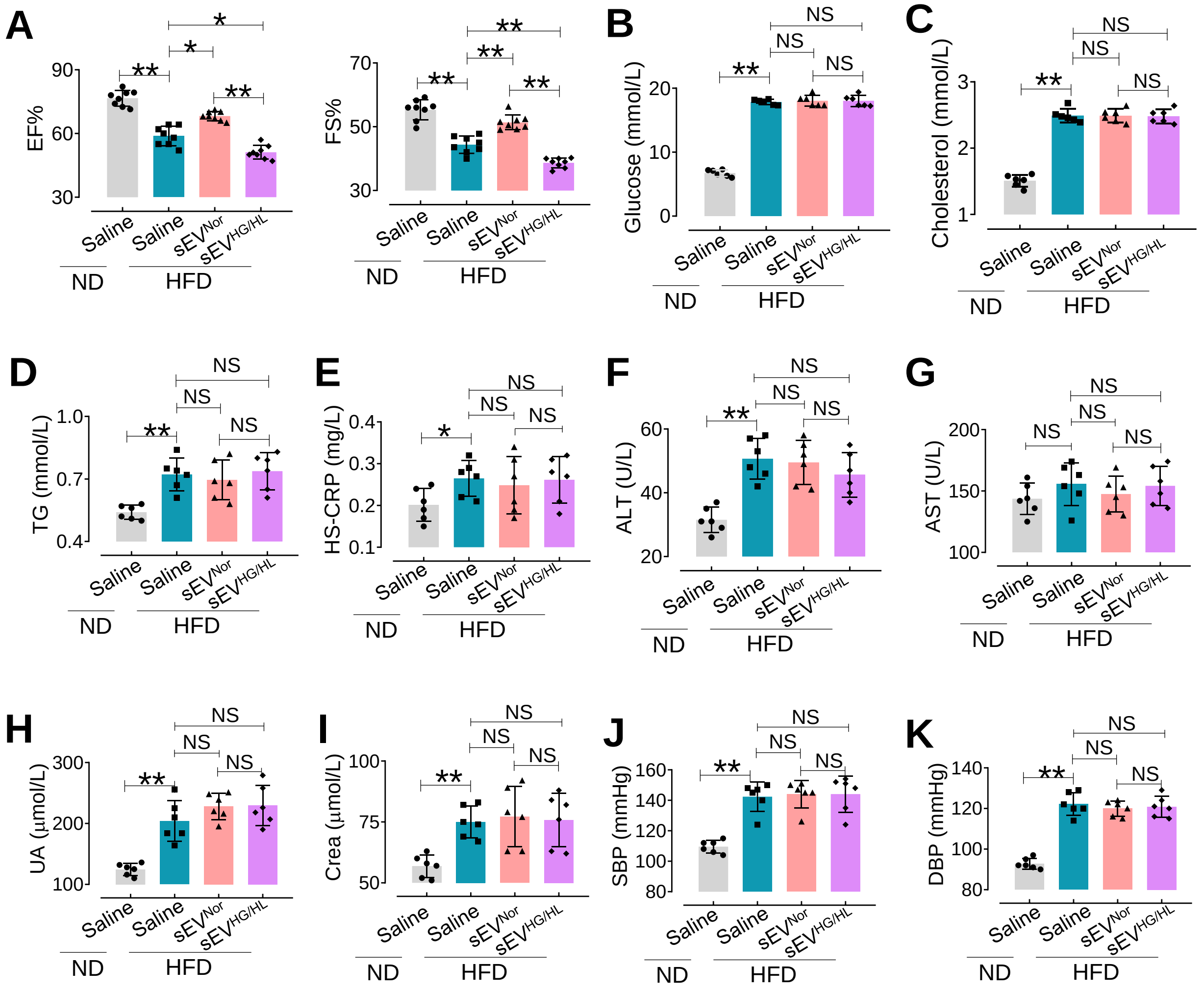

Supplementary Figure 4. Cardiac ultrasound and blood biochemical parameters of HFD mice treated with Myo-sEVs derived from the same number of cardiomyocytes. (A-K) Ejection fraction (EF) and fraction shortening (FS) (A) and physiological and biochemical indexes including serum glucose (B), cholesterol (C), triglyceride (TG, D), HS-CRP (E), ALT (F), AST (G), UA (H), Crea (I), blood pressure (J-K) in HFD mice treated with Myo-sEVs^{Nor} or Myo-sEVs$^{HG/HL}$ delivered from the same number of cardiomyocytes. (n = 6-8). All values are presented as mean ± SEM. P values were calculated by one-way ANOVA, Tukey's multiple comparisons test. **p < 0.01.

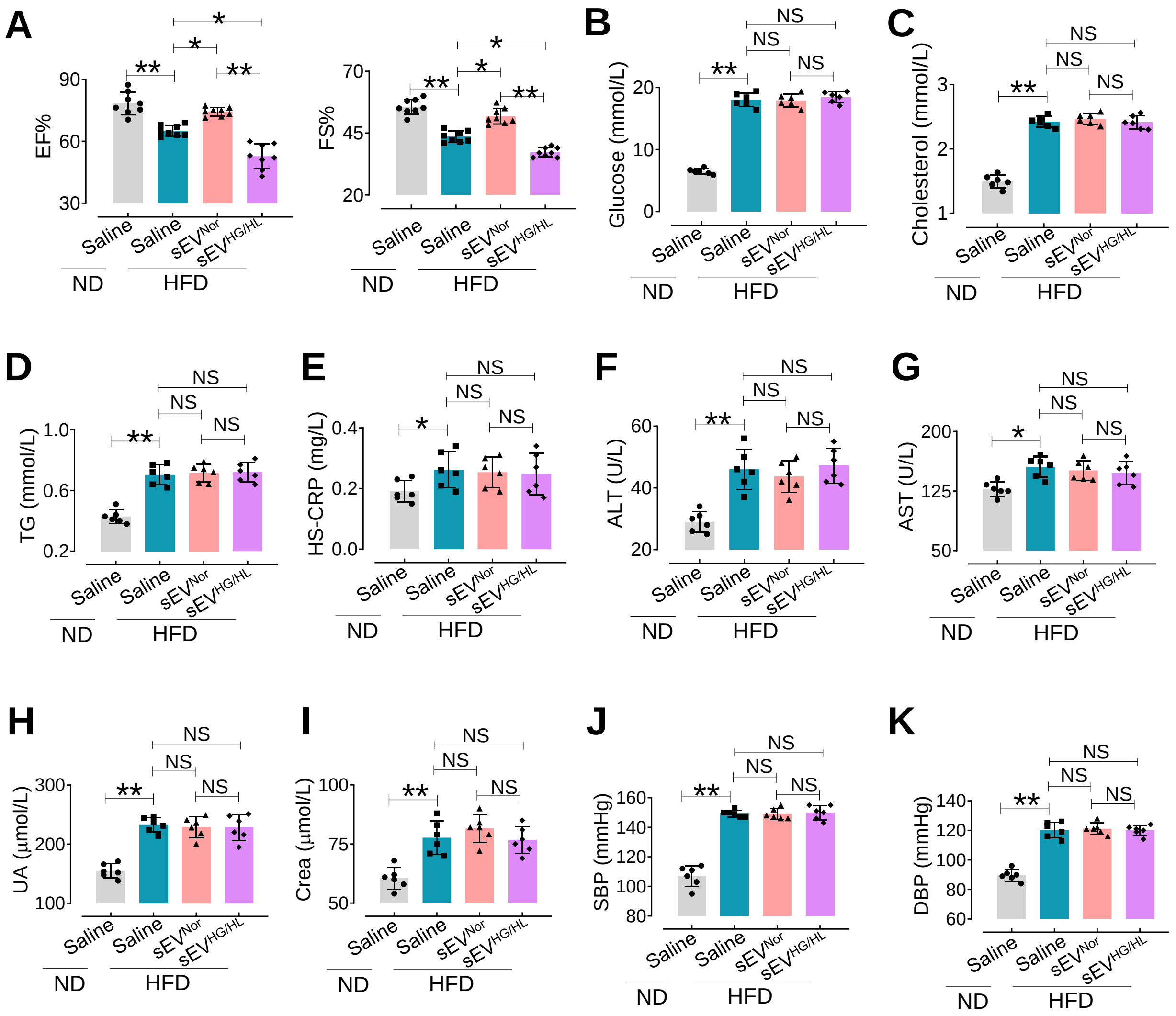

Supplementary Figure 5. Cardiac ultrasound and blood biochemical parameters of HFD mice treated with the same number of indicated Myo-sEVs. (A-K) Ejection fraction (EF) and fraction shortening (FS) (A) and physiological and biochemical indexes including serum glucose (B), cholesterol (C), triglyceride (TG, D), HS-CRP (E), ALT (F), AST (G), UA (H), Crea (I), blood pressure (J-K) in HFD mice treated with the same number of Myo-sEVs^{Nor} or Myo-sEVs$^{HG/HL}$. ($n = 6$-8). All values are presented as mean ± SEM. P values were calculated by one-way ANOVA, Tukey's multiple comparisons test. *$p < 0.05$, **$p < 0.01$.

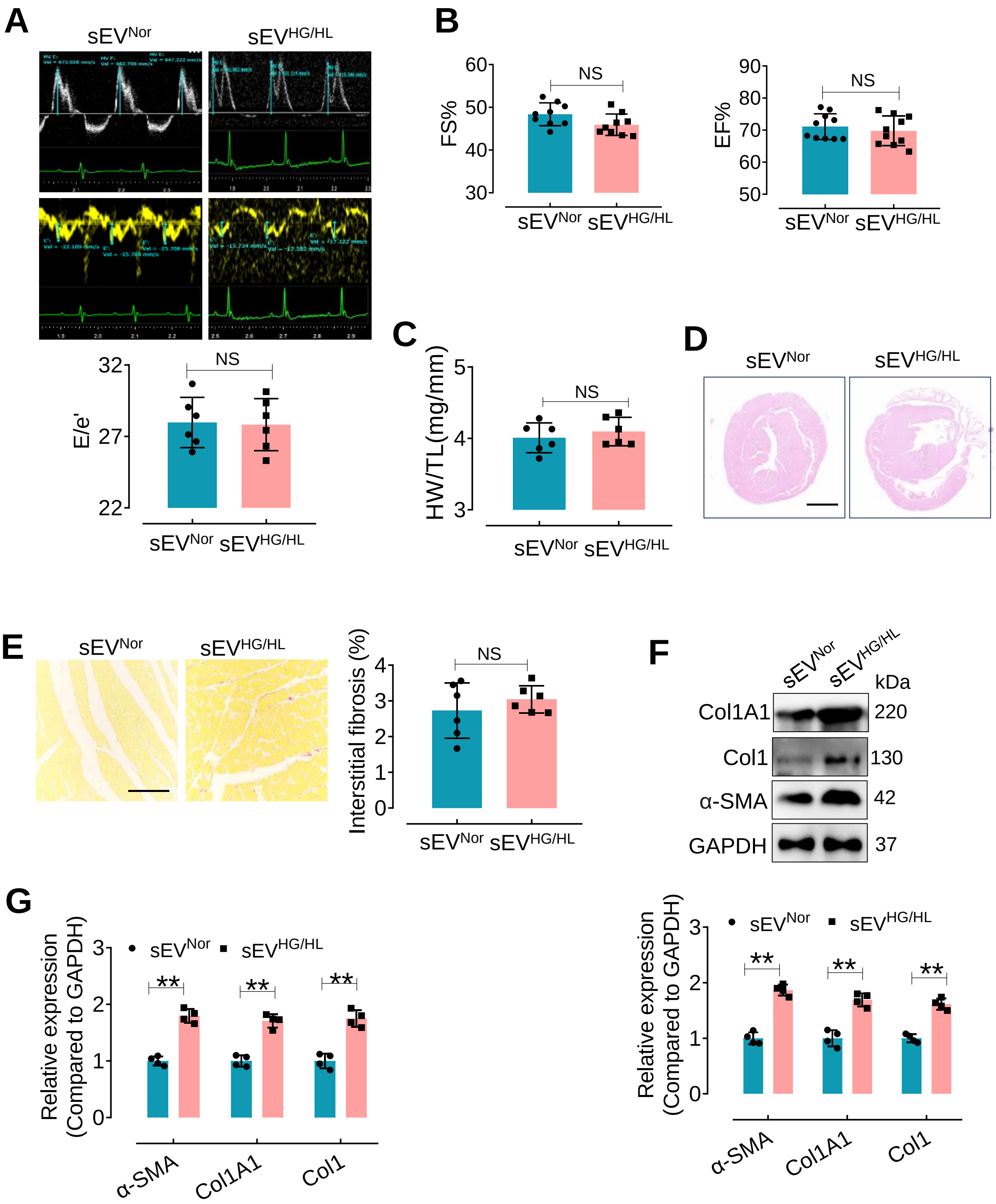
A
sEV^Nor
sEV^HG/HL
E/e'
32
27
22
NS
sEV^Nor sEV^HG/HL
B
FS%
60
50
40
30
NS
sEV^Nor sEV^HG/HL
EF%
90
80
70
60
50
NS
sEV^Nor sEV^HG/HL
C
HW/TL(mg/mm)
5
4
3
NS
sEV^Nor sEV^HG/HL
D
sEV^Nor
sEV^HG/HL
E
sEV^Nor
sEV^HG/HL
Interstitial fibrosis (%)
4
3
2
1
0
NS
sEV^Nor sEV^HG/HL
F
sEV^Nor
sEV^HG/HL
kDa
Col1A1
220
Col1
130
α-SMA
42
GAPDH
37
G
Relative expression (Compared to GAPDH)
3
2
1
0
sEV^Nor
sEV^HG/HL
**
**
**
α-SMA
Col1A1
Col1
Relative expression (Compared to GAPDH)
3
2
1
0
sEV^Nor
sEV^HG/HL
**
**
**
α-SMA
Col1A1
Col1

Supplementary Figure 6. Myo-sEVs$^{HG/HL}$ promote fibrotic marker protein expression but don't affect cardiac diastolic function and myocardial fibrosis in normal C57BL/6 mice. (A) E-waves (upper panel) were measured using pulsed wave (PW) Doppler from a 4-chamber view of the lateral mitral valve. Early diastolic (e') velocities were obtained from the Tissue Doppler signal of the mitral annulus (lower panel). The ratio of peak E to peak e' was not changed in normal C57BL/6 mice after treatment with Myo-sEVs$^{HG/HL}$. (n = 6). **(B)** Ejection fraction (EF) and fraction shortening (FS) of normal C57BL/6 mice after treatment with Myo-sEVs$^{HG/HL}$ or Myo-sEVs^{Nor}. (n = 8-10). **(C)** No significant alteration in the hearts and tibia length (mm) ratio (HW/TL) in normal C57BL/6 mice upon treatment with Myo-sEVs $^{HG/HL}$ compared to Myo-sEVs^{Nor}. (n = 6). **(D)** Hematoxylin and eosin staining of whole mouse heart histological sections were performed after *in vivo* treatment of normal mice using Myo-sEV^{Nor} and Myo-sEV$^{HG/HL}$. Scale bar: 1 mm. (**E)** Representative images of histological sections of mouse hearts and quantification of Picrosirius red-stained areas showing collagen deposition after *in vivo* treatment of normal mice with Myo-sEV^{Nor} and Myo-sEV$^{HG/HL}$ (n = 6). Scale bars: 100 μm. **(F, G)** The protein (F) and mRNA (G) expression levels of Col1α1, Col1, α-SMA were measured through Western blotting and qPCR separately in the hearts of normal mice with indicated treatment (n = 4). All values are presented as mean ± SEM. P values were calculated by unpaired two-tailed Student's t-test. **p < 0.01.

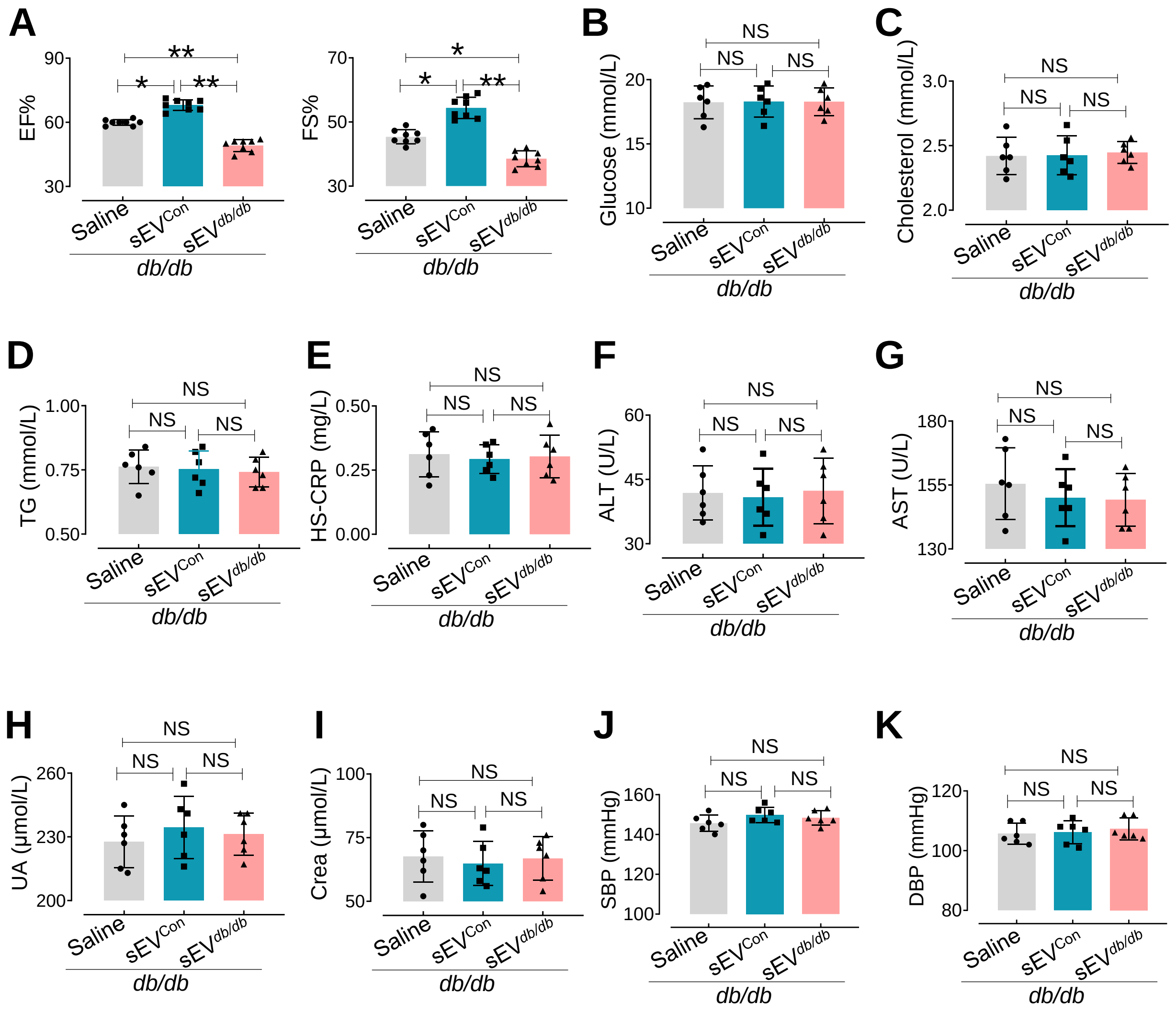

Supplementary Figure 7. Cardiac ultrasound and blood biochemical parameters of *db/db* mice treated with the same number of indicated Myo-sEVs. (A-K) Ejection fraction (EF) and fraction shortening (FS) (A) and physiological and biochemical indexes including serum glucose (B), cholesterol (C), triglyceride (TG, D), HS-CRP (E), ALT (F), AST (G), UA (H), Crea (I), blood pressure (J-K) in HFD mice treated with Myo-sEVs^{Nor} or Myo-sEVs$^{HG/HL}$ delivered from the same number of cardiomyocytes. (n = 6-8). All values are presented as mean ± SEM. P values were calculated by one-way ANOVA, Tukey's multiple comparisons test. *p < 0.05, **p < 0.01.

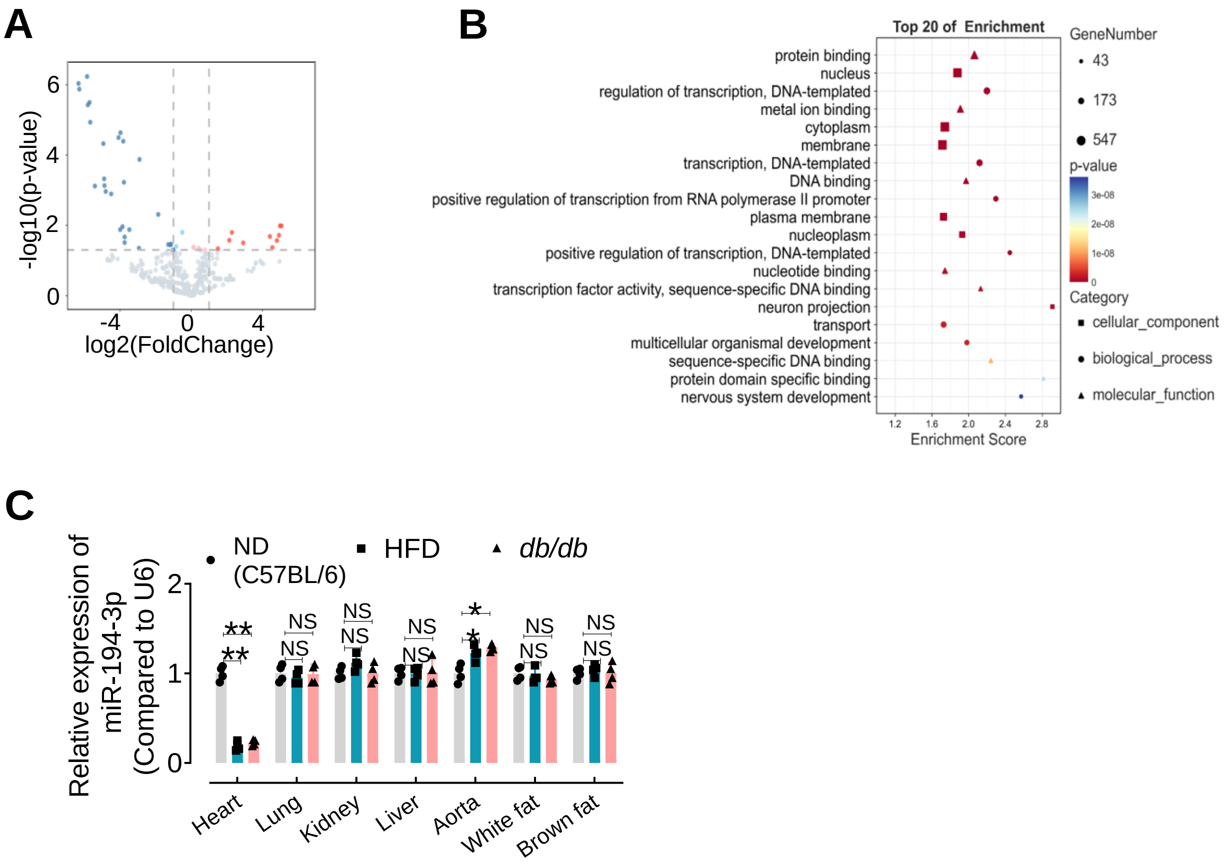

Supplementary Figure 8. Bioinformatics analysis of Myo-sEV microRNAs with differential expression. **(A)** Volcanic map analysis showed the miRNAs with different levels in Myo-sEVs^{Nor} and Myo-sEVs $^{HG/HL}$. **(B)** Go map analysis showed the categories of predicted target genes of 11 differentially expressed Myo-sEV miRNAs with identical sequences in both humans and mouse. (**C**) The relative expression of miR-194-3p was measured in various tissues, including the heart, lung, kidney, liver, aorta, white fat, and brown fat from normal diet (ND), high-fat diet (HFD), and *db/db* mice (n = 4). All values are presented as mean ± SEM. P values were calculated by one-way ANOVA, Tukey's multiple comparisons test. *$p < 0.05$, **$p < 0.01$.

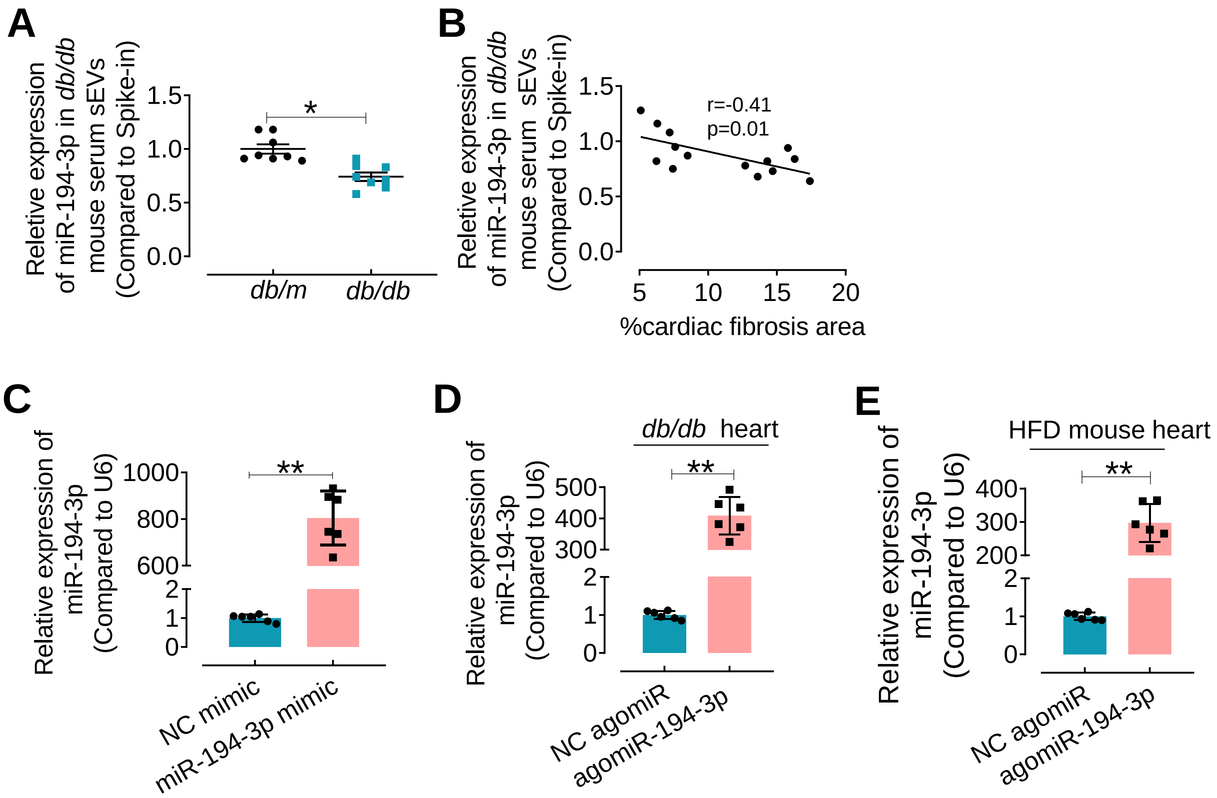

Supplementary Figure 9. miR-194-3p levels in serum sEVs and CD172a^{+} serum sEVs in HFD mice treated with the same number of Myo-sEVs^{Nor} or Myo-sEVs$^{HG/HL}$. (**A**) The relative expression of miR-194-3p was detected by qPCR in serum sEVs in HFD mice treated with the same number of Myo-sEV^{Nor} or Myo-sEV$^{HG/HL}$ (cel-miR-39 miRNA as a spike-in control, n = 8). (**B**) miR-194-3p levels in serum sEVs displayed a negative correlation with alterations in the cardiac fibrosis area, as determined by Spearman's correlation analysis. (n = 14). (**C**) miR-194-3p levels in fibroblasts treated with miR-194-3p mimic or negative control (NC mimic). (n = 6). (**D, E**) The relative expression of miR-194-3p in the hearts from *db/db* (D) or HFD mice (E) treated with agomiR-194-3p or negative control (NC). (n = 6). All values are presented as mean ± SEM. P values were calculated by unpaired two-tailed Student's t-test. *p < 0.05, **p < 0.01.

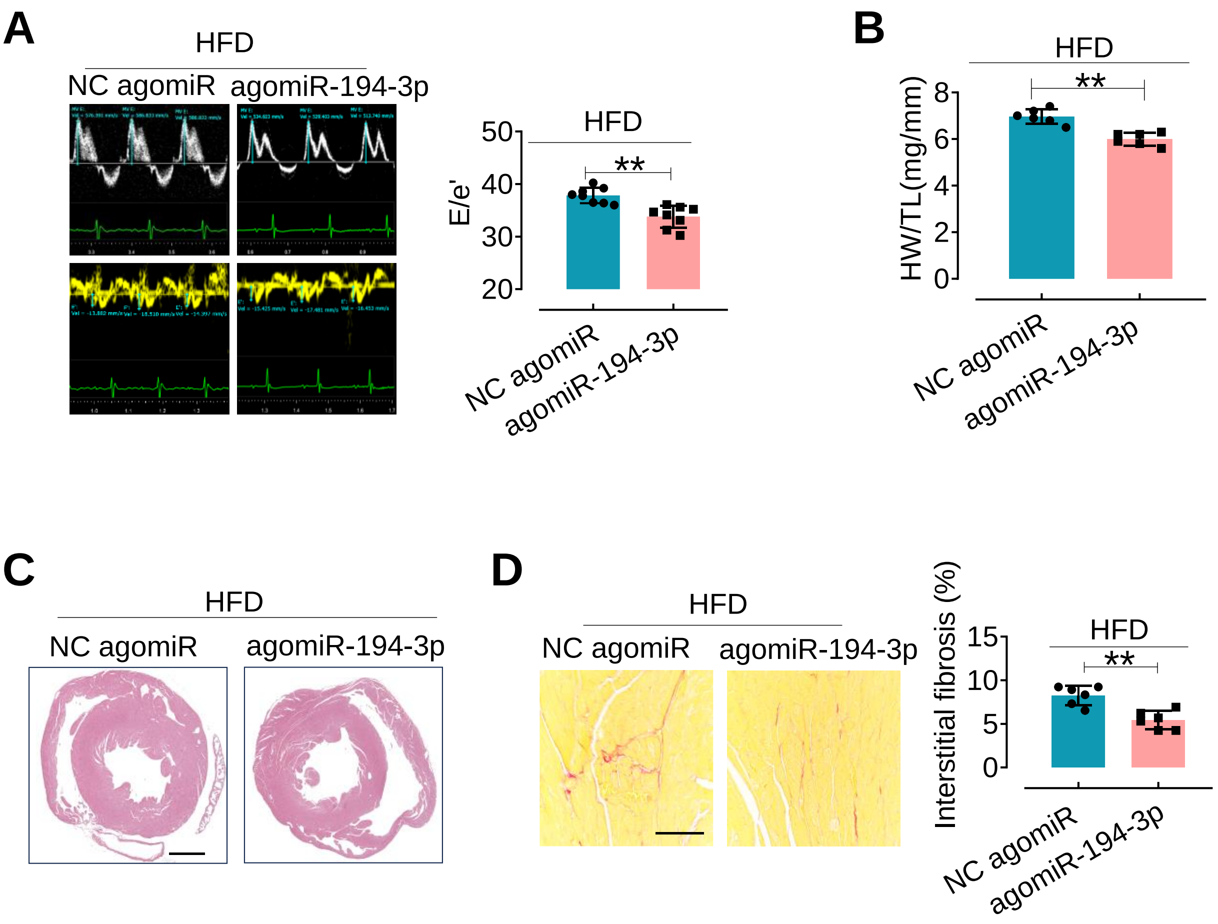

Supplementary Figure 10. agomiR-194-3p reverses myocardial fibrosis in HFD mice. **(A)** The E to e' peak ratio significantly decreased in the hearts of HFD mice following in vivo treatment with agomiR-194-3p. (n = 8). **(B)** A decreased hearts and tibia length (mm) ratio (HW/TL) was observed in HFD mice upon treatment with agomiR-194-3p compared to those treated with NC. (n = 6). **(C)** Representative images showed in whole mouse heart histological sections stained with hematoxylin and eosin. Scale bars: 1 mm. **(D)** Picrosirius red-staining showing collagen deposition in mouse hearts after the HFD mice *in vivo* treated with agomiR-194-3p and NC. Scale bars: 100 μm. (n = 6). All values are presented as mean ± SEM. P values were calculated by unpaired two-tailed Student's t-test. $*p < 0.05$, $**p < 0.01$.

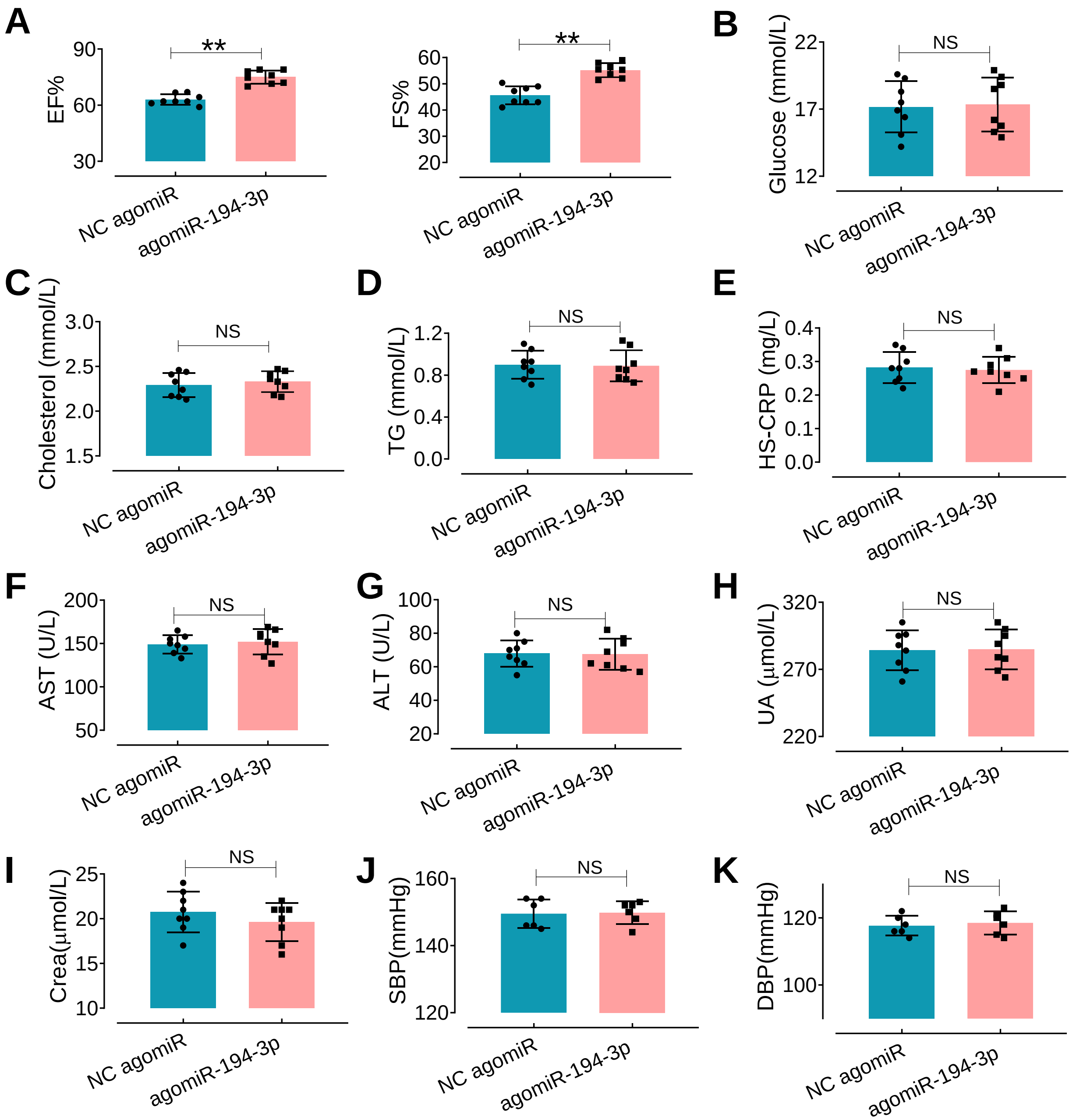

Supplementary Figure 11. Cardiac ultrasound and blood biochemical parameters of *db/db* mice treated with agomiR-194-3p. (A-K) Ejection fraction (EF) and fraction shortening (FS) (A), and physiological and biochemical indexes including serum glucose (B), cholesterol (C), triglyceride (TG, D), HS-CRP (E), AST (F), ALT (G), UA (H), Crea (I), blood pressure (J-K) in *db/db* mice treated with NC or agomiR-194-3p. (n = 6-8). All values are presented as mean ± SEM. P values were calculated by unpaired two-tailed Student’s t-test. *p < 0.05, **p < 0.01.

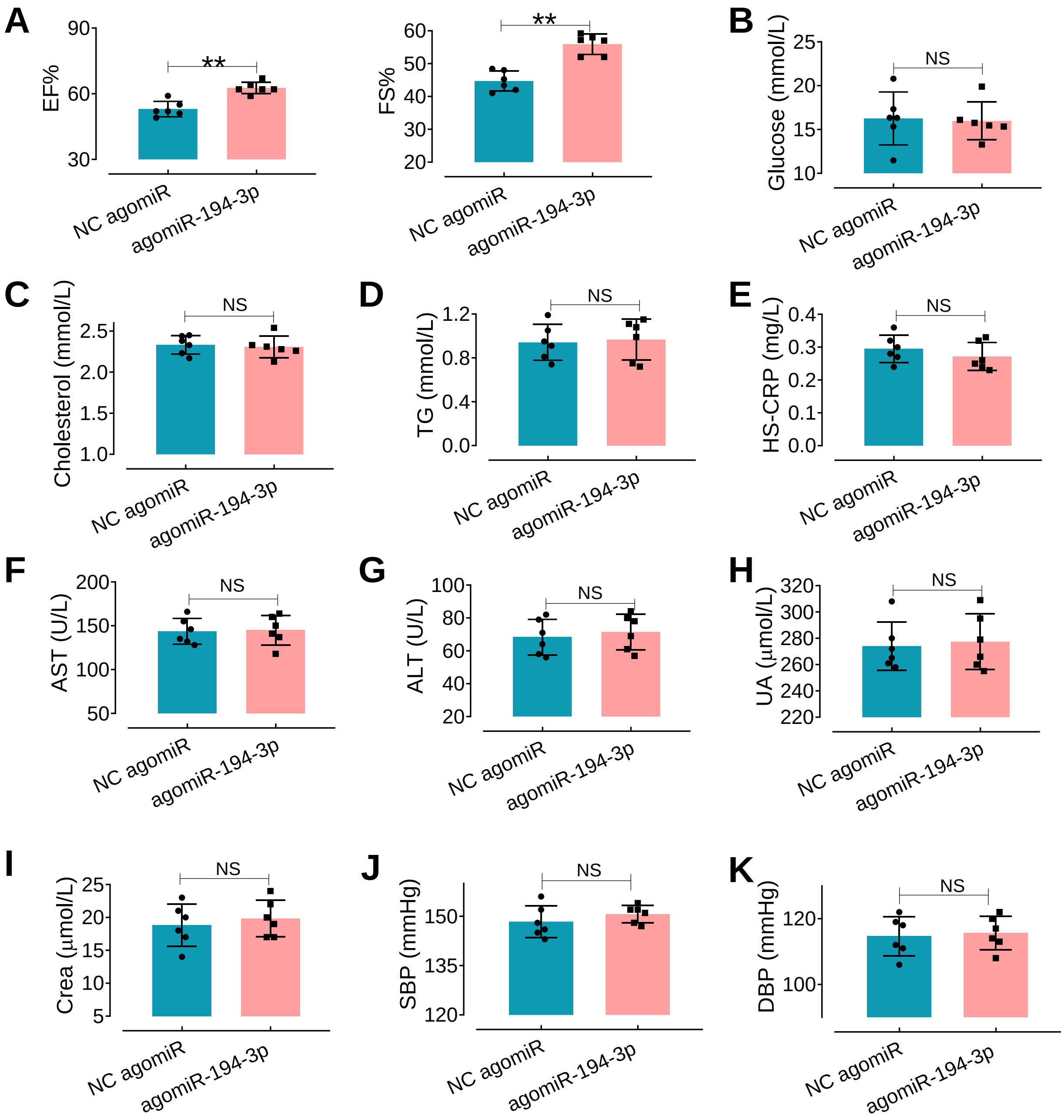

Supplementary Figure 12. Cardiac ultrasound and blood biochemical parameters of HFD mice treated with agomiR-194-3p. (A-K) Ejection fraction (EF) and fraction shortening (FS) (A), and physiological and biochemical indexes including serum glucose (B), cholesterol (C), triglyceride (TG, D), HS-CRP (E), AST (F), ALT (G), UA (H), Crea (I), blood pressure (J-K) in HFD mice treated with NC or agomiR-194-3p. (n = 6). All values are presented as mean ± SEM. P values were calculated by unpaired two-tailed Student's t-test. $*p < 0.05$, $**p < 0.01$.

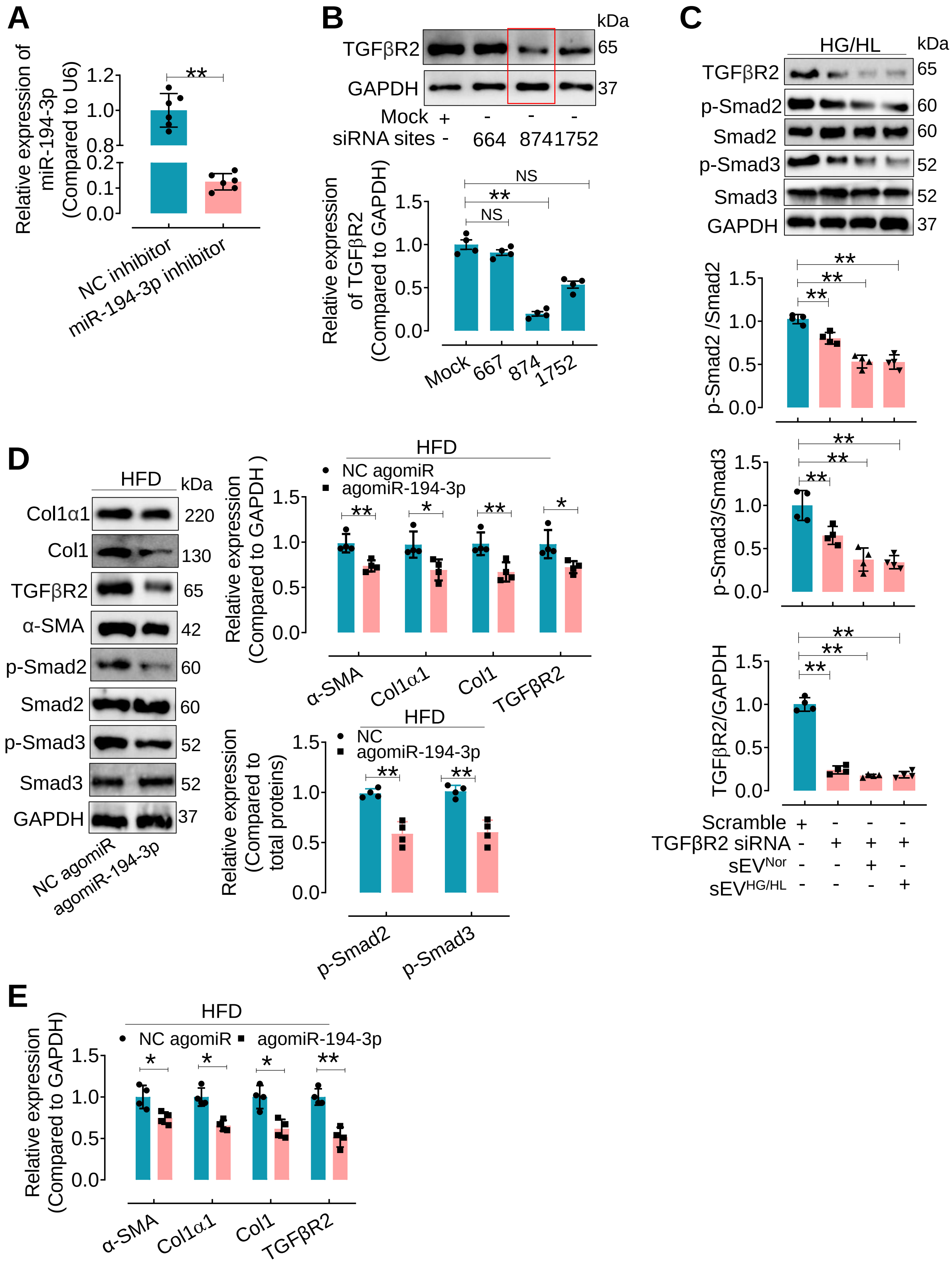
A
Relative expression of miR-194-3p (Compared to U6)
NC inhibitor
miR-194-3p inhibitor
B
TGFβR2
GAPDH
kDa
65
37
Mock
siRNA sites
664
8741752
Relative expression of TGFβR2 (Compared to GAPDH)
NS
Mock
667
874
1752
C
HG/HL
TGFβR2
p-Smad2
Smad2
p-Smad3
Smad3
GAPDH
p-Smad2 /Smad2
p-Smad3/Smad3
TGFβR2/GAPDH
Scramble
TGFβR2 siRNA
sEVNor
sEVHG/HL
D
HFD
Col1α1
Col1
α-SMA
NC agomiR
agomiR-194-3p
Relative expression (Compared to GAPDH)
Relative expresion (Compared to total proteins)
NC
E

Figure S13. The upregulated expression of TGFβR2 by Myo-sEVs$^{HG/HL}$ or diabetes was reversed by TGFβR2 siRNA or agomiR-194-3p. (A) The relative expression of miR-194-3p in fibroblasts treated with miR-194-3p inhibitor or negative control (NC inhibitor) (n = 6). **(B)** The relative expression of TGFβR2 in fibroblasts treated with 3 different TGFβR2 siRNAs. (n = 4). **(C)** Western blotting analysis of TGFβ signaling pathway-related molecules was performed in cardiac fibroblasts after 24 h treatment of Myo-sEV $^{HG/HL}$ and TGFβR2 siRNA. (n = 4). **(D-E)** Expression levels of Col1α1, Col1, α-SMA, and TGFβ signaling pathway-related molecules were measured by Western blotting (D) or qPCR (E) assay in heart tissue of HFD mice treated with agomiR-194-3p and negative control (NC). (n = 4). All values are presented as mean ± SEM. P values were calculated by unpaired two-tailed Student's t-test (A, B, D, E) or one-way ANOVA followed by Tukey's test (C). *p < 0.05, **p < 0.01.

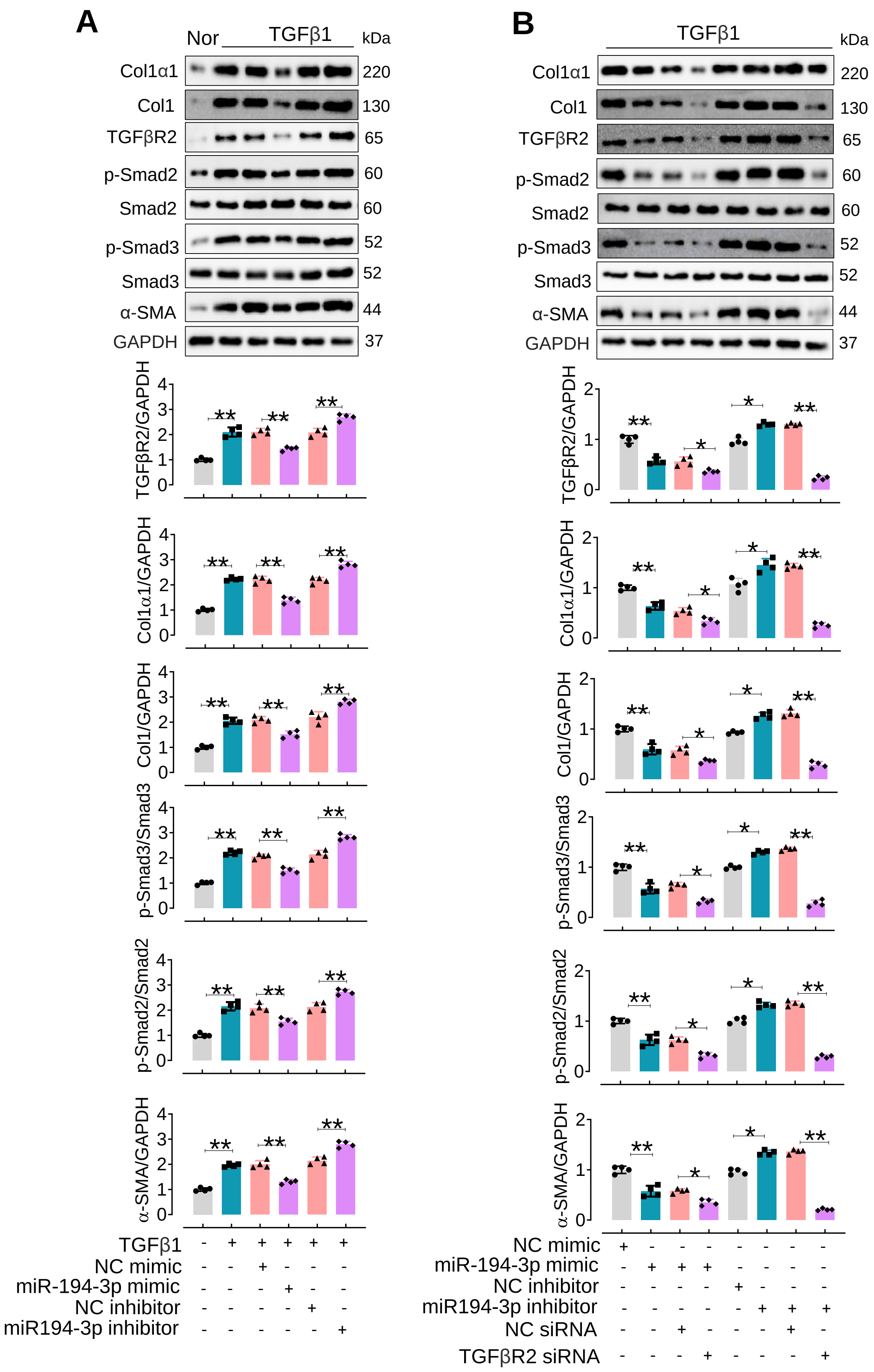
A
Nor
TGFβ1
kDa
Col1α1
Col1
TGFβR2
p-Smad2
Smad2
p-Smad3
Smad3
α-SMA
GAPDH
220
130
65
60
60
52
52
44
37
TGFβR2/GAPDH
Col1α1/GAPDH
Col1/GAPDH
p-Smad3/Smad3
p-Smad2/Smad2
α-SMA/GAPDH
TGFβ1
NC mimic
miR-194-3p mimic
NC inhibitor
miR194-3p inhibitor
B
NC siRNA
TGFβR2 siRNA

Figure S14. The upregulated expression of the TGFβ signaling pathway and related markers of myocardial fibrosis was reversed by TGFβR2 siRNA or agomiR-194-3p. (A-B) Expression levels of Col1α1, Col1, α-SMA, and TGFβ signaling pathway-related molecules were measured by Western blotting in cardiac fibroblasts after 24 h treatment with TGFβ1(10 ng/mL, MCE, China), miR-194-3p mimic or miR-194-3p inhibitor, TGFβR2 siRNA. ($n = 4$). All values are presented as mean ± SEM. P values were calculated by one-way ANOVA followed by Tukey's test (A-B). *$p < 0.05$, **$p < 0.01$.

Supplementary Table 1. Clinical and biochemical characteristics of T2DM patients and non-T2DM individuals in the study.

	non-T2DM （n = 30）	T2DM （n = 30）	*p* value
Gender (Males/Females)	20/20	21/19	0.501
Age (years)	48.77 ± 5.81	51.13 ± 5.96	0.059
BMI (kg/m^2)	23.03 ±1.46	26.81 ± 1.23	< 0.001
Fasting plasma glucose (mmol/L)	5.06 ± 0.54	7.32 ± 0.89	< 0.001
Fasting Insulin (μU/ml)	6.76 ± 1.22	11.85 ± 2.92	< 0.001
HbA1c (%)	4.99 ± 0.47	7.89 ± 1.1	< 0.001
Triglycerides (mmol/L)	1.09 ± 0.25	1.67 ± 0.20	< 0.001
Cholesterol (mmol/L)	3.46 ± 0.98	4.14 ± 1.1	0.013
HDL cholesterol (mmol/L)	1.37 ± 0.17	1.24 ± 0.21	0.017
LDL cholesterol (mmol/L)	2.083 ± 0.43	2.29 ± 0.38	0.057
Systolic blood pressure (mmHg)	113.4 ± 6.47	116.2 ± 8.15	0.15
Diastolic blood pressure (mmHg)	72.37 ± 5.84	71.1 ± 4.52	0.35

Statistical significance was calculated by unpaired two-tailed Student’s t-test of non-T2DM vs. T2DM patients. All values are given as mean ± SEM. n.s.-not significant.

Abbreviations: BMI, Body Mass Index; HbA1c, Glycated hemoglobin A1c.

Supplementary Table 2. The antibodies used in the study.

Name	Company	Item No.
CD63 antibody	SBI	EXOAB-CD63A-1
CD81 antibody	SBI	EXOAB-CD81A-1
TSG101 Antibody	Abcam	ab125011
Alix antibody	Abcam	ab117600
Calnexin antibody	Abcam	ab92573
Albumin antibody	Abcam	ab207327
α-sarcomeric actin antibody	Abcam	ab9465
α-SMA antibody	Abcam	ab7817
Collagen 1 antibody	Abcam	ab260043
Vimentin antibody	Abcam	ab8978
TGFβ1 antibody	Abcam	ab215715
TGFβ2 antibody	Abcam	ab53778
TGFβR1 antibody	Abcam	ab235578
TGFβR2 antibody	Abcam	ab259360
TGFβR3 antibody	Abcam	ab166705
Col1α1 antibody	Cell signal technology	#72026
Smad2/3 Antibody Kit	Cell signal technology	#12747
GAPDH antibody	Cell signal technology	#5174
Feeder cells antibody	Miltenyi Biotec	130-120-802
CD31 antibody	ABclonal	A23703
CD45 antibody	ABclonal	A23707
Cardiac troponin T antibody	ABclonal	A4914

Supplementary Table 3. Primers used in the RT-PCR assays.

Name	5'-3' sequence Forward primer	Reverse primer	Products
α-SMA	GCTGGACTCTGGAGATGG	GCAGTAGTCACGAAGGAATGA	182bp
BMP7	CTCTGGCCAGCCTGCAAGATA	CCGGAACTCTCGATGGTGGA	147bp
BMPr1	CCTGTTCGTCGTGTCTCAT	CTGGTGCTAAGGTTACTCC	116bp
BMPr2	GAGCCCTCCCTGGACTTG	ATATCGACCCCGTCCAATC	103bp
Col1α1	TAGGCCATTGTGTATGCAGC	ACATGTTCAGCTTTGTGGACC	190bp
Col7α1	CGCCAAGAGATGAGTCAGCAC	CTCTGCAGGTAGGGCAGGGT	147bp
CDC25a	AGAACCCTATTGTGCCTACTG	TACTCATTGCCGAGCCTATC	123bp
CDK14	TGTCAGTACATGGACAAGCACCC T	TGTAAGACAGACCTCGCAGCAA CT	163bp
CD36	AAAGTCACTGCGACATGATTAAT GG	AACGTCGGATTCAAATACAGCA TAG	174bp
CDK6	TGCACAGTGTCACGAACAGAC	TGAATGAAAAGCCTGCCTGGG	151bp
Collagen1	GGAGAGTACTGGATCGACCCTAA C	CTGACCTGTCTCCATGTTGCA	169bp
GAPDH	CGTGCCGCCTGGAGAAACC	TGGAAGAGTGGGAGTTGCTGTT G	174bp
HGF	CCGACGAGACCGAATCAATAAC	CGTCCCTTTATAGCTGCCTCC	189bp
Irak1	GCACCCACAACTTCTCGGAG	CACCGTGTTCCTCATCACCG	128bp
Ptpn12	CCTTACTTACGCTTCATT	GGGCAGTGGCTTTATTTT	153bp
Smad2	GTTCCTGCCTTTGCTGAC	TCTCTTTGCCAGGAATGCTT	142bp
Slc26a	CCTGGATATTGCAAAGCCTTCAG	GAATCCTGGGAAGGGTCAAAGC	520bp
TAK1	AGAGGAGCCTTTGGAGTTGT	CCATCACAAGACACACTGGA	138bp
Tab2	GCAGCAAAGGAACATCTAGCC	TGGACTGTTAAGTACAGGTGGA	146bp
TGFβ1	ATCCTGTCCAAACTAAGGCTCG	GATGGCGTTGTTGCGGTC	155bp
TGFβ2	CCATCCCGCCCACTTTCT	CCTCCGCTCTGGTTTTCACA	194bp
TGFβR1	AATCAAGTGTGGAGCAACATG	AGCCCTGTATTCCGTCTCCT	201bp
TGFβR2	CAGCTGTGCAAGTTTTGCGA	TTCTGCGGCTTCTCACAGAT	189bp
TGFβR3	CACATCCTAAACCTCCGCAGTA	ACAGAACCCTCCGAAACCAG	175bp
Pri-miR-539	CGTGAGGTTGTCATGCTGAGT	TCATCCCGGAAGTCCACC	294bp
Pri-miR-143	CGGATAGGAGGCAGACCA	AGGAACATCGCCACAAGC	266bp
Pri-miR-181-1	CAGAGACTATGTTTACAAAGGTC AC	CAGAAATAGGACTTGATATGAA TTG	236bp
Pri-miR-181-2	TCATTGCTGTCGGTGGGT	GGTCCCACCTGGAGCAGTA	135bp
Pri-miR-194	ATCTGGGGTGGCGGCTA	TTATGGCTGGCATTGAGGTG	238bp
Pri-miR-574	GGAACCGTCCCGAGAAAG	TGGCTTGTGCCTAGCCTCT	217bp

Supplementary Table 4. Echocardiographic parameters of HFD mice after tail vein injection with Myo-sEVs$^{HG/HL}$ or Myo-sEVs^{Nor}.

Echo parameters	ND mice	HFD mice		
	Saline (n = 8)	Saline (n = 8)	sEV^{Nor} (n = 8)	sEV$^{G/HL}$ (n = 8)
Heart rate (ms)	450.4 ± 19.5	437.8 ± 20.6	448.3 ± 26.1	445.3 ± 24.2
LVEDV (mL)	68.41 ± 20.13	74.55 ± 17.33	70.32 ± 11.52	71.45 ± 9.87
LVESV (mL)	40.21 ± 6.9	74.36 ± 8.25	62.73 ± 7.78$^{\#}$	89.11 ± 11.96*
EDD (mm)	3.7 ± 0.42	3.96 ± 0.48	3.82 ± 0.33	3.71 ± 0.39
ESD (mm)	2.46 ± 0.39	3.51 ± 0.52	2.7 ± 0.51$^{\#}$	4.68 ± 0.53*
Peak E (cm/sec)	429.34 ± 58.32	544.89 ± 50.33	652.3 ± 44.23$^{\#}$	901.5 ± 31.48*
Peak A (cm/sec)	300.21 ± 44.21	275.82 ± 37.32	326.5 ± 31.86	360.8 ± 45.93
E/A ratio	1.43 ± 0.34	2.18 ± 0.11	2.09 ± 0.07$^{\#}$	2.48 ± 0.16*
e' (cm/sec)	-16.65 ± 10.45	-16.34 ± 12.05	-17.77 ± 11.56	-31.91 ± 12.43
a' (cm/sec)	-15.63 ± 11.93	-15.89 ± 11.71	-16.28 ± 14.46	-16.73 ± 13.71
IVRT (msec)	14.35 ± 2.14	11.45 ± 0.54	12.84 ± 1.79$^{\#}$	9.79 ± 0.92*
IVCT (msec)	10.54 ± 1.45	13.47 ±1.32	12.1 ± 2.06$^{\#}$	14.99 ± 1.58*
LVET (msec)	54.65 ± 3.21	45.23 ± 3.75	49.86 ± 5.12$^{\#}$	42.94 ± 4.02*
LA; d (mm)	2.14 ± 0.53	2.43 ± 0.47	2.34 ± 0.43	2.48 ± 0.51
LA; s (mm)	0.63 ± 0.18	1.14 ± 0.25	0.91 ± 0.21$^{\#}$	1.28 ± 0.15*

Statistical significance was calculated by two-way ANOVA, and Tukey's multiple comparisons test. All values are given as mean ± SEM. *refers to a comparison between sEV^{Nor}-treated or sEV$^{HG/HL}$-treated HFD mice (* $P < 0.05$), # refers to a comparison between saline-treated or sEV^{Nor}-treated HFD mice (# $P < 0.05$).

Abbreviations: LVEDV, Left Ventricle End Diastolic Volume; LVESV, Left Ventricle End Systolic Volume; EDD, End Diastolic Diameter; ESD, End Systolic Diameter; E, Early mitral inflow diastole peak; A, Late mitral inflow diastole peak; e', Early annular diastole peak; a', Late annular diastole peak; IVRT, Isovolumetric Relaxation Time; IVCT, Isovolumetric Contraction Time; LVET, Left Ventricular Ejection Time; LA; d, Left Atrial diastolic size; LA; s, Left Atrial systolic size.

Supplementary Table 5. 38 miRNAs with different expressions in Myo-sEVs^{Nor} and Myo-sEVs$^{HG/HL}$ identified by miRNA chip assay.

No.	Name	Log2 (Fold_Change)	Regulation	*p*.value
1	mmu-miR-5134-5p	-6.3413	down	9.18E-07
2	mmu-miR-365-2-5p	-6.29272	down	1.33E-06
3	mmu-miR-1947-5p	-5.85733	down	5.83E-07
4	mmu-miR-7658-5p	-5.81312	down	3.74E-06
5	mmu-miR-7051-3p	-5.72097	down	3.18E-06
6	mmu-miR-6409	-5.67983	down	1.16E-05
7	mmu-miR-539-5p	-5.41743	down	7.60E-04
8	mmu-miR-3970	-4.94163	down	4.72E-05
**9**	**mmu-miR-194-3p**	**-4.89644**	**down**	**4.73E-04**
10	mmu-miR-92b-5p	-4.87958	down	7.37E-04
11	mmu-miR-448-5p	-4.80875	down	0.001084
12	mmu-miR-6908-3p	-4.50154	down	0.001273
13	mmu-miR-143-5p	-4.08979	down	3.17E-05
14	mmu-miR-6939-5p	-3.99543	down	0.013192
15	mmu-miR-3089-3p	-3.97929	down	2.31E-05
16	mmu-miR-1198-5p	-3.86293	down	0.010844
17	mmu-miR-6970-3p	-3.82232	down	4.03E-05
18	mmu-miR-669a-5p	-3.78867	down	5.92E-04
19	mmu-miR-8090	-3.74641	down	0.030775
20	mmu-miR-6980-3p	-3.74599	down	0.021426
21	mmu-miR-7653-3p	-3.48167	down	0.013188
22	mmu-let-7f-1-3p	-2.93931	down	0.044504
23	mmu-miR-181b-5p	-2.90924	down	1.33E-04
24	mmu-miR-704	-1.85531	down	0.004881
25	mmu-miR-7018-5p	-1.29202	down	0.035001
26	mmu-miR-7030-5p	-1.16867	down	0.035885
27	mmu-miR-7038-5p	-1.14351	down	0.03397
28	mmu-miR-214-5p	-1.00467	down	0.049603
29	mmu-miR-7080-3p	1.499927	up	0.046112
30	mmu-miR-6973b-5p	2.14094	up	0.026527
31	mmu-miR-30c-1-3p	2.285495	up	0.015935
32	mmu-miR-706	2.924041	up	0.031476

33	mmu-miR-466q	4.424656	up	0.020626
34	mmu-miR-466i-3p	4.562108	up	0.042837
35	mmu-miR-669p-3p	4.817545	up	0.027014
36	mmu-miR-466f-3p	4.934275	up	0.019135
37	mmu-miR-574-3p	4.991257	up	0.010314
38	mmu-miR-466g	5.077568	up	0.010272

Statistical significance was calculated by unpaired two-tailed Student's t-test of Myo-sEVs^{Nor} and Myo-sEVs$^{HG/HL}$. (n = 3).

Supplementary Table 6. Echocardiographic parameters of HFD mice treated with agomiR-194-3p.

Echo parameters	HFD		
	NC agomiR (n = 6)	agomiR-194-3p (n = 6)	*p*.value
Heart rate (ms)	441.5 ± 17.87	442.4 ± 19.02	n.s.
LVEDV (mL)	69.71 ± 11.61	69.02 ± 12.05	n.s.
LVESV (mL)	70.52 ± 9.82	42.92 ± 9.44	< 0.01
EDD (mm)	4.012 ± 0.59	3.65 ± 0.45	n.s.
ESD (mm)	3.44 ± 0.51	2.54 ± 0.55	< 0.01
Peak E (cm/sec)	668.3 ± 29.44	594.5 ± 19.15*	< 0.01
Peak A (cm/sec)	313.2 ± 22,56	331.2 ± 25.26	n.s.
E/A ratio	2.06 ± 0.06	1.7 ± 0.1*	< 0.01
e' (cm/sec)	-17.86 ± 14.13	-33.3 ± 15.41	n.s.
a' (cm/sec)	-18.84 ± 12.52	-17.37 ± 11.42	n.s.
IVRT (msec)	11.84 ± 1.86	15.21 ± 1.52*	< 0.01
IVCT (msec)	12.67 ± 1.7	10.7 ± 1.65*	< 0.05
LVET (msec)	42.86 ± 5.27	55.04 ± 5.03*	< 0.01
LA;d (mm)	2.28 ± 0.45	2.08 ± 0.33	n.s.
LA;s (mm)	1.2 ± 0.19	0.79 ± 0.18*	< 0.01

Statistical significance was calculated by unpaired two-tailed Student’s t-test of mock vs. agomiR-194-3p mice. All values are given as mean ± SEM. n.s., not significant.

Abbreviations: LVEDV, Left Ventricle End Diastolic Volume; LVESV, Left Ventricle End Systolic Volume; EDD, End Diastolic Diameter; ESD, End Systolic Diameter; E, Early mitral inflow diastole peak; A, Late mitral inflow diastole peak; e’, Early annular diastole peak; a’, Late annular diastole peak; IVRT, Isovolumetric Relaxation Time; IVCT, Isovolumetric Contraction Time; LVET, Left Ventricular Ejection Time; LA; d, Left Atrial diastolic size; LA; s, Left Atrial systolic size.

References

1. American Diabetes Association Professional Practice C. 2. Classification and Diagnosis of Diabetes: Standards of Medical Care in Diabetes-2022. Diabetes Care. 2022; 45: S17-S38.
2. Marino F, Salerno N, Scalise M, Salerno L, Torella A, Molinaro C, et al. Streptozotocin-Induced Type 1 and 2 Diabetes Mellitus Mouse Models Show Different Functional, Cellular and Molecular Patterns of Diabetic Cardiomyopathy. Int J Mol Sci. 2023; 24.
3. Mohamed TMA, Ang YS, Radzinsky E, Zhou P, Huang Y, Elfenbein A, et al. Regulation of Cell Cycle to Stimulate Adult Cardiomyocyte Proliferation and Cardiac Regeneration. Cell. 2018; 173: 104-16 e12.
4. Ackers-Johnson M, Li PY, Holmes AP, O'Brien SM, Pavlovic D, Foo RS. A Simplified, Langendorff-Free Method for Concomitant Isolation of Viable Cardiac Myocytes and Nonmyocytes From the Adult Mouse Heart. Circ Res. 2016; 119: 909-20.
5. Gan L, Xie D, Liu J, Bond Lau W, Christopher TA, Lopez B, et al. Small Extracellular Microvesicles Mediated Pathological Communications Between Dysfunctional Adipocytes and Cardiomyocytes as a Novel Mechanism Exacerbating Ischemia/Reperfusion Injury in Diabetic Mice. Circulation. 2020; 141: 968-83.
6. Nicks AM, Holman SR, Chan AY, Tsang M, Young PE, Humphreys DT, et al. Standardised method for cardiomyocyte isolation and purification from individual murine neonatal, infant, and adult hearts. J Mol Cell Cardiol. 2022; 170: 47-59.
7. Sano S, Horitani K, Ogawa H, Halvardson J, Chavkin NW, Wang Y, et al. Hematopoietic loss of Y chromosome leads to cardiac fibrosis and heart failure mortality. Science. 2022; 377: 292-7.
8. Thery C, Amigorena S, Raposo G, Clayton A. Isolation and characterization of exosomes from cell culture supernatants and biological fluids. Curr Protoc Cell Biol. 2006; Chapter 3: Unit 3 22.
9. Gao E, Lei YH, Shang X, Huang ZM, Zuo L, Boucher M, et al. A novel and efficient model of coronary artery ligation and myocardial infarction in the mouse. Circ Res. 2010; 107: 1445-53.
